# Supplementary material for: The E3 ubiquitin ligase WWP2 regulates pro-fibrogenic monocyte infiltration and activity in heart fibrosis
Source: Nat Commun. 2022 Nov 30;13:7375. doi: 10.1038/s41467-022-34971-6 (PMC9712659; doi:10.1038/s41467-022-34971-6)
Supplement: Supplementary file 1 — Supplementary Information [file 41467_2022_34971_MOESM1_ESM.pdf]

SUPPLEMENTARY INFORMATIONS

The E3 ubiquitin ligase WWP2 regulates pro-fibrogenic monocyte infiltration and activity in heart fibrosis

Huimei Chen[1,2,#,*], Gabriel Chew[1,#], Nithya Devapragash[1], Jui Zhi Loh[1], Kevin Y Huang[1], Jing Guo[1], Shiyang Liu[1], Elisabeth Li Sa Tan[1], Shuang Chen[2,3], Nicole Gui Zhen Tee[4], Masum M Mia[1], Manvendra K Singh[1], Aihua Zhang[3], Jacques Behmoaras[1,5,*] and Enrico Petretto[1,2,6,*]

[1]Programme in Cardiovascular and Metabolic Disorders, Duke-NUS Medical School, 8 College Road, 169857 Singapore
[2]Institute for Big Data and Artificial Intelligence in Medicine, School of Science, China Pharmaceutical University, Nanjing, 210009 China
[3]Department of Nephrology, Children's Hospital of Nanjing Medical University, Nanjing, 210008 China
[4]National Heart Centre Singapore, 169609 Singapore
[5]Centre for Inflammatory Disease, Imperial College London, Hammersmith Hospital, London, W12 0NN UK
[6]MRC London Institute of Medical Sciences (LMC), Imperial College, Faculty of Medicine, London W12 0NN UK

[#], equal contribution

*Correspondence to Huimei Chen (huimei.chen@duke-nus.edu.sg), Jacques Behmoaras (jacquesb@duke-nus.edu.sg) or Enrico Petretto (enrico.petretto@duke-nus.edu.sg)
Duke-NUS Medical School, Programme in Cardiovascular and Metabolic Disorders, 8 College Road 169857 Singapore, Republic of Singapore Tel: (65) 6601 5114

Supplementary Figure 1

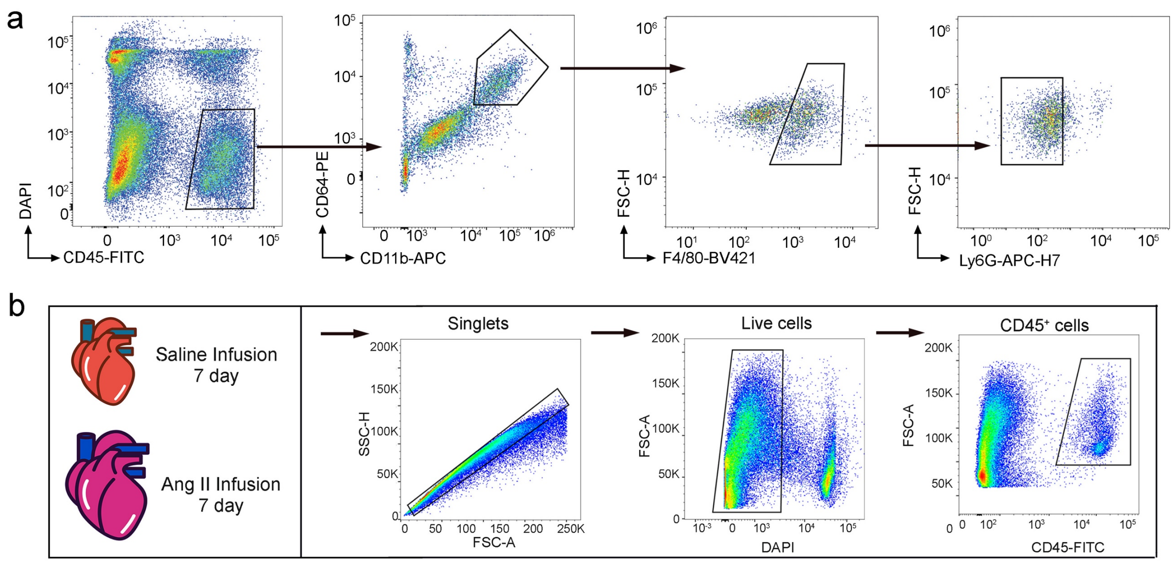

Supplementary Figure 1. a. Flow cytometry gating strategy for cardiac macrophages. Representative flow cytometry plots of the gating scheme used to characterize total cardiac macrophages (Live, $CD45^{+}CD64^{+}CD11b^{+}F4/80^{+}Ly6G^{-}$). **b.** Graphical representation of the experimental setup for scRNA-seq sample preparation. Samples were isolated from hearts (left ventricle, LV) of Ang II-infused (500ng/kg/min, 7 days) or sham-operated C57BL6/J male mice 1 week after surgery. LVs were digested and $CD45^{+}$ live cells were FACS-sorted and analyzed by scRNA-seq.

Supplementary Figure 2

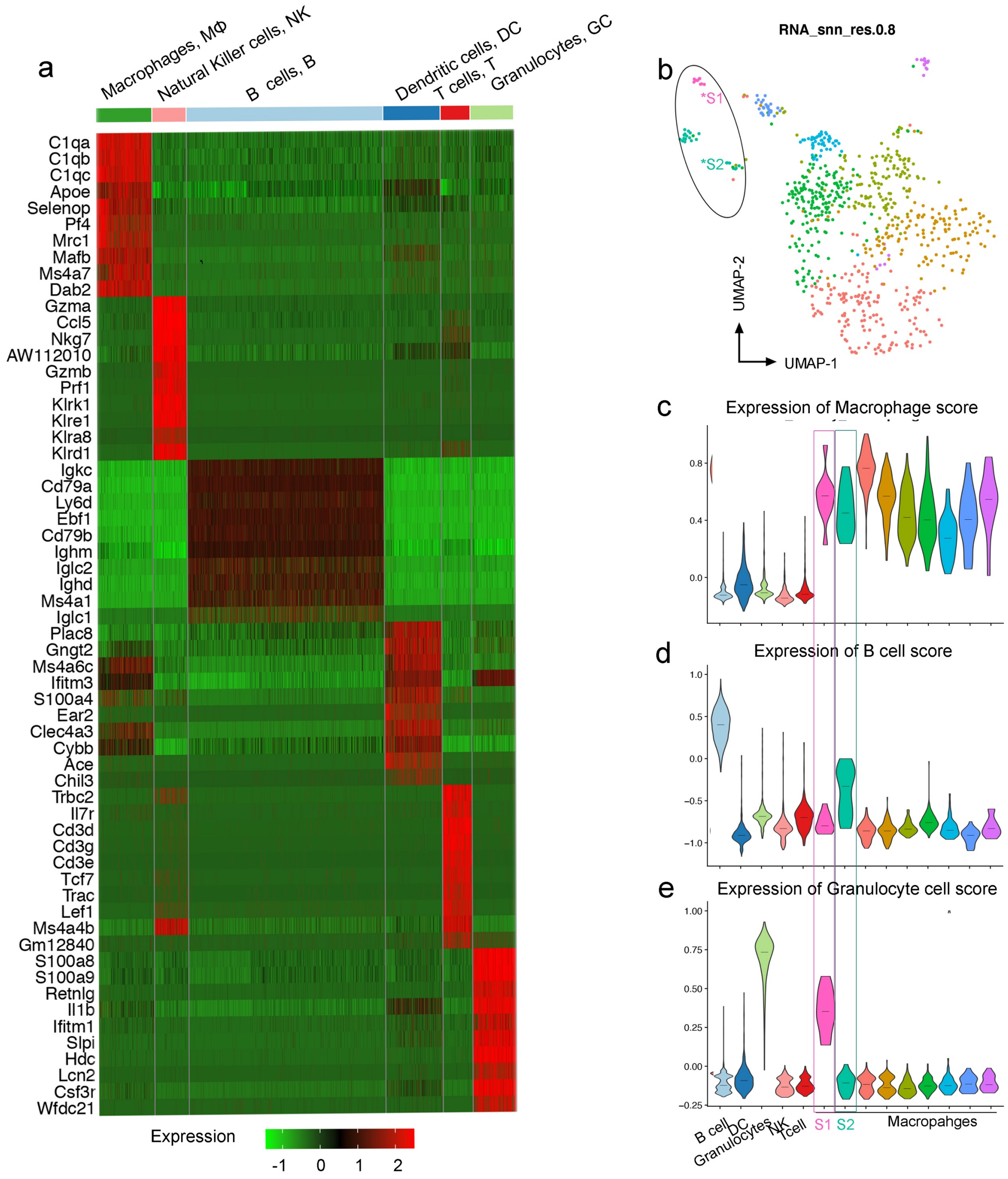

Supplementary Figure 2. Single cell RNA sequencing (scRNA-seq) profiling of CD45+ immune cells in hearts. a. Heatmap of scaled gene expression of the top 10 differentially expressed genes (DEGs) ordered by $\log_2$ fold change (FC) in cardiac CD45+ cardiac immune cells from scRNA-seq analysis of WT-saline and WT-Ang CD45+ cells as described in Figure 1b. **b.** Uniform manifold approximation and projection (UMAP) of macrophage clusters including outlier clusters (circled as S1 and S2). **c-e.** Violin plots of cell type expression scores for c) macrophages, d) B-cells, and e) granulocytes based on marker genes derived from *Skelley et al*. [1]. Scores are shown for CD45+ immune cell types (granulocytes, NK Cells, DC, macrophages, T-cells, and B-cells). The small clusters S1 and S2 (highlighted) showed relative high enrichment for granulocytes and B-cells markers, respectively, despite strong enrichment for macrophage markers, and were excluded from analysis.

Supplementary Figure 3

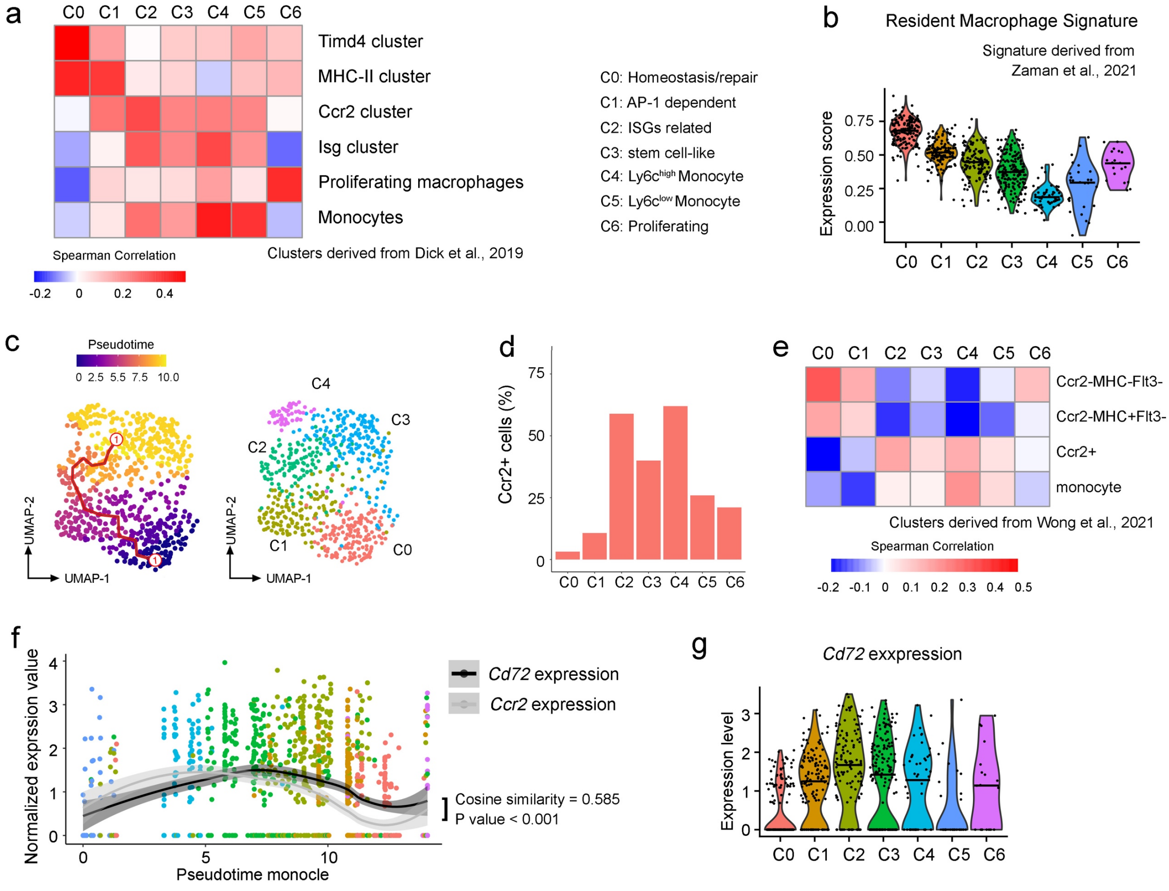

Supplementary Figure 3. ScRNA-seq analysis reveals distinct cardiac macrophages in NICM a. Spearman correlation of specificity matrices between macrophage clusters from the NICM murine model (this study, **Figure 1d)** and macrophage subpopulations isolated following myocardial infarction (MI, 11 days) from Dick et al. [2] (see Methods for details) **b.** Violin plots of resident macrophage signature across macrophage subclusters identified from hearts after treatment with Ang-II infusion, derived from a previously published study Zaman et al. [3]. **c.** UMAP of cardiac macrophages with single-cell trajectories from C0 to C3 (*left,* red line) superimposed to single-cell clusters (*right*). The main trajectories are generated by Monocle analysis, and cell colors are determined by the average pseudotime over all cells. **d.** Percentage of Ccr2+ cells in WT macrophage clusters following Ang II infusion (500ng/kg/min, 7 days). **e.** Spearman correlation of specificity matrices between macrophage clusters from NICM murine model (this study, **Figure 1d)** and *Ccr2*$^{+/-}$ macrophage subpopulations from genetic murine model of dilated cardiomyopathy (DCM) [4] (see Methods for details) **f.** Normalized expression of *Cd72* (black line) and *Ccr2* (grey line) in macrophages from scRNA-seq analysis in LV from WT Ang II-treated (500ng/kg/min, 7 days) mice across monocle-derived pseudotime trajectory inferred in main **Figure 1g**. Cosine similarity was quantified to infer similarity between *Cd72* and *Ccr2* mRNA expression patterns. **g.** Violin plot of *Cd72* mRNA expression across all macrophage subclusters identified in **Figure 1c**.

Supplementary Figure 4

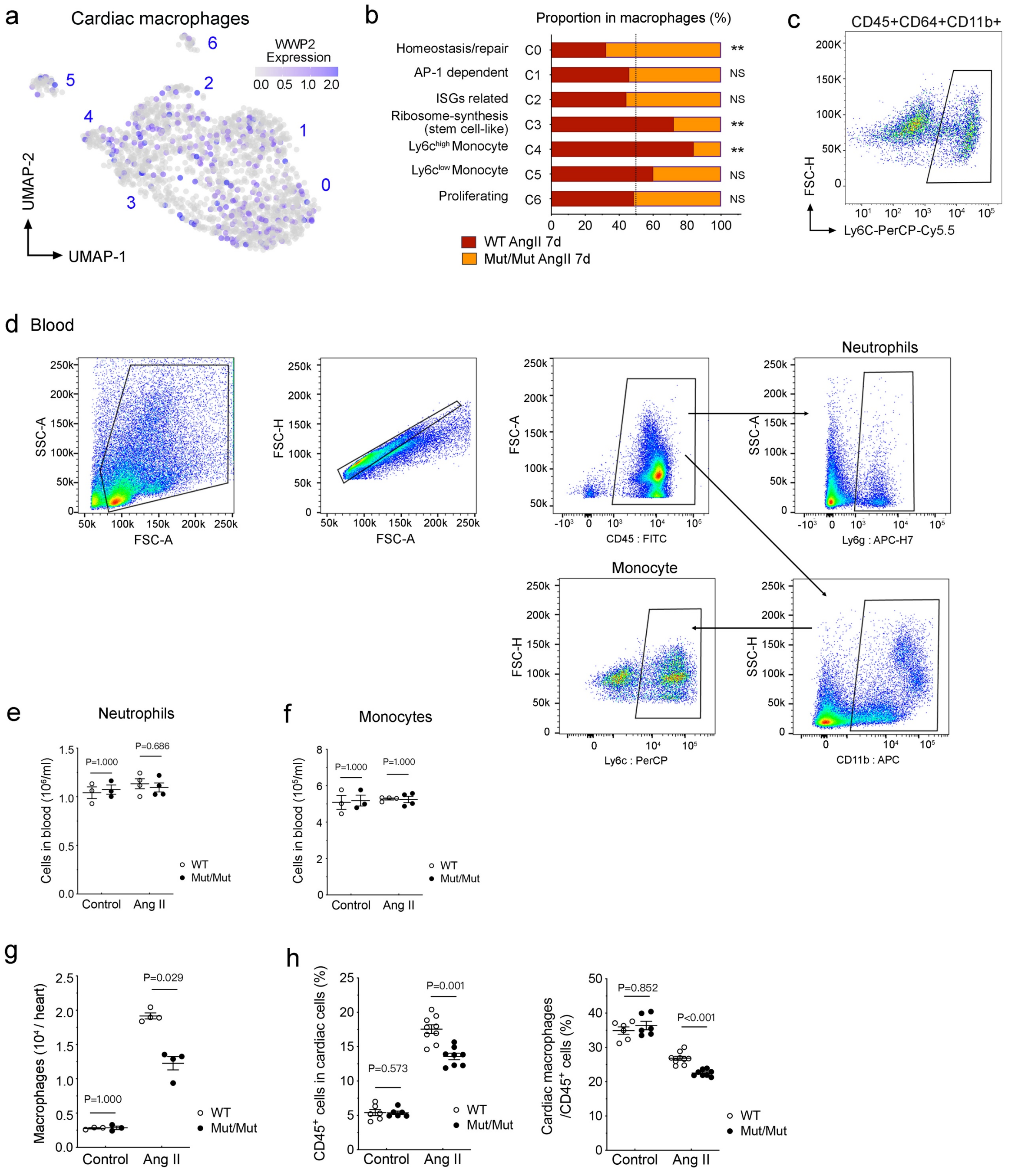

Supplementary Figure 4. *Wwp2* mRNA expression, macrophage cell proportions, macrophage gating, and other details of macrophage and blood cells in our model. a. Uniform manifold approximation and projection (UMAP) plots of normalized expression of *Wwp2* in WT and WT-Ang II (500ng/kg/min, 7 days) macrophages (related to **Figure 1c**). **b.** The proportion of different macrophage clusters in WT and WWP2$^{Mut/Mut}$ mice upon Ang II infusion (500ng/kg/min, 7 days). **c.** Representative flow cytometry plot showing the sorting of Ly6C^{high} population in cardiac monocyte compartment (CD45+CD64+CD11b+). **d.** Representative flow cytometry plots showing the gating strategy used in characterizing neutrophils and monocytes (Live, CD45^{+} Ly6G^{+} and live, CD45+ CD11b^{+} Ly6C^{+}) in blood. **e-f.**

Quantification analysis of absolute neutrophil (**e**) and monocyte (**f**) numbers in blood (/ml) derived from WT and WWP2$^{Mut/Mut}$ mice with or without Ang II infusion (500ng/kg/min, 7 days; n=4-5 for each group, statistical significance calculated by non-parametric Mann-Whitney U test, data reported as mean ± SD). **g.** The absolute cardiac macrophage numbers in WT and WWP2$^{Mut/Mut}$ mice with or without Ang II infusion (500ng/kg/min, 7 days). n=3-4 for each group, statistical significance calculated by non-parametric Mann-Whitney U test, data are shown as dot-plots with mean ± SD. **h.** Percentage of cardiac CD45^{+} cells (*left*) and macrophages (*right*) in CD45^{+} cells (live, CD45^{+}CD64^{+}CD11b^{+}F4/80^{+}Ly6G^{-}) isolated from WWP2$^{Mut/Mut}$ hearts (left ventricle) with respect to WT with or without Ang II infusion (500ng/kg/min, 7 days); n=6 for each group, statistical significance calculated by non-parametric Mann-Whitney U test, data are shown as dot-plots with mean ± SD**.**

Supplementary Figure 5

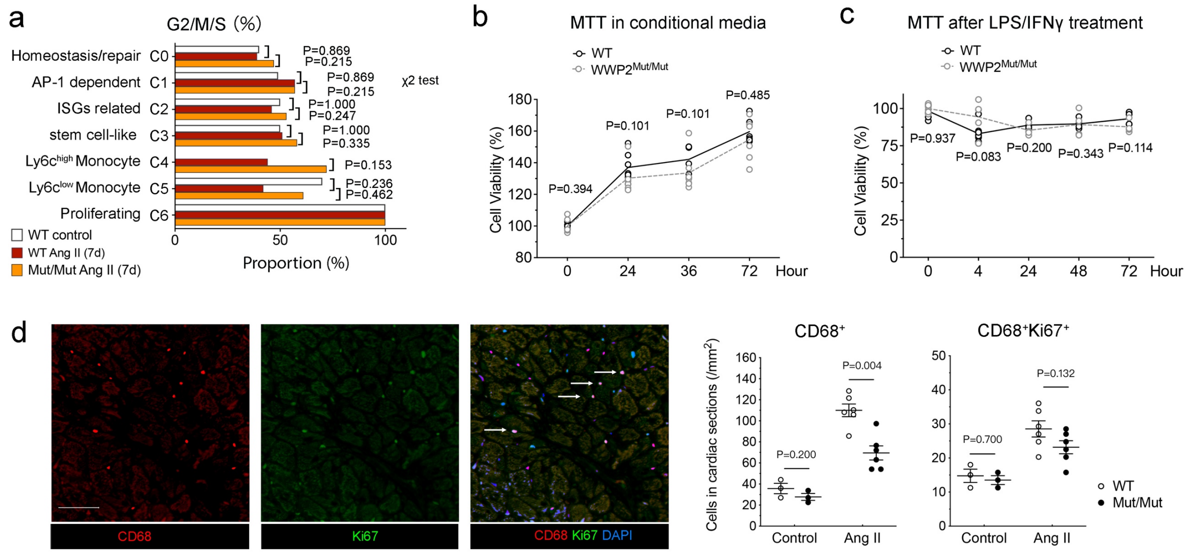

Supplementary Figure 5. Proliferation and migration data in WT and WWP2$^{Mut/Mut}$ macrophages. a. Percentages of cardiac macrophages in G2/M/S phase in experimental groups (WT control, WT post Ang-II infusion, and WWP2$^{Mut/Mut}$ post Ang-II infusion) for clusters 0, 1, 2, 3, 5, 6, and 7. Ang II treatment, 500ng/kg/min, 7 days. **b-c.** MTT (3-[4,5-dimethylthiazol-2-yl]-2,5 diphenyl tetrazolium bromide) assay showing the relative cell viability in unstimulated WT and WWP2$^{Mut/Mut}$ BMDMs (**b**) and in LPS/IFNɣ conditional media (**c**) (100ng/ml; 10ng/ml, 4 hrs) at different time points (hours) (statistical significance calculated by non-parametric Mann-Whitney U test, WWP2$^{Mut/Mut}$ vs WT group, n=4-9 for each group). **d.** Photomicrographs of immunohistochemical staining of CD68 and Ki-67 in myocardial heart (left ventricle) sections from mice after Ang II-infusion (500ng/kg/min, 7 days) (*left*). Size bar indicates 50 μm. Arrows indicate representative CD68^{+}Ki67^{+} cells. Quantification of CD68^{+} and CD68^{+}Ki67^{+} cells (*right*). Data obtained from 5 visual field/section and 3 sections per mice were averaged and plotted in controls and Ang II treated mice. Statistical significance calculated by non-parametric Mann-Whitney U test, n=3-6 for each group; data are shown as dot-plots with mean ± SD.

Supplementary Figure 6

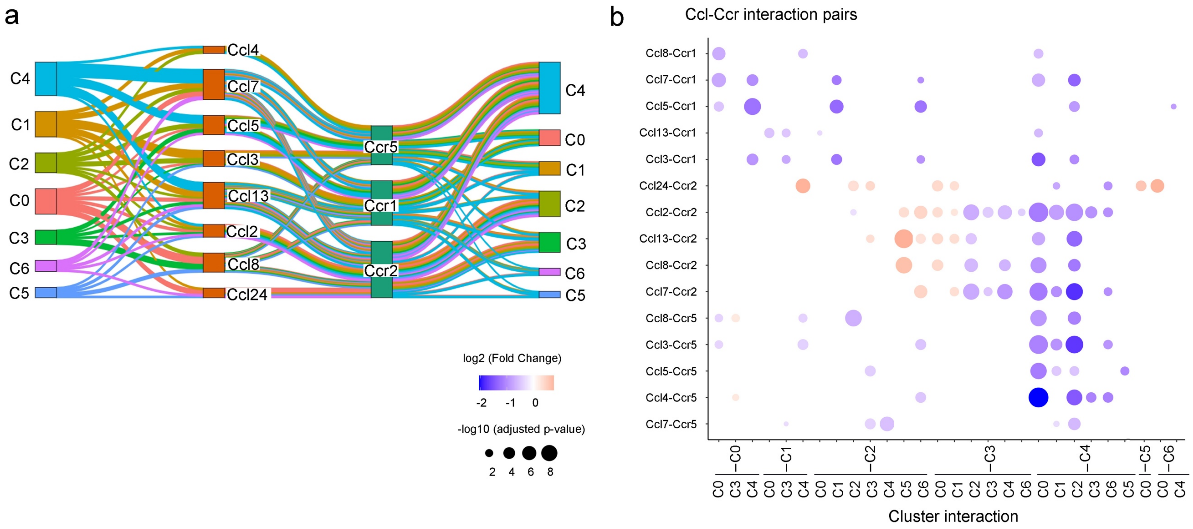

Supplementary Figure 6. Ligand/receptor analysis in subsets of cardiac macrophages during NICM. a. Sankey diagram of significant chemokine ligand-receptor interactions between macrophage sub-clusters identified by scRNA-seq analysis. The height of rectangles corresponds to the number of interactions involving the ligand, receptor for each macrophage cluster. **b.** Bubble plot of significantly differentially expressed (WWP2$^{Mut/Mut}$ *vs* WT post Ang II-infusion (500ng/kg/min, 7 days)) chemokine ligand-receptor pairs with respect to specific receptor macrophage cluster and ligand macrophage cluster (*bottom*: Ligand; *top*: Receptor). Log_2 fold change (FC) is represented by colour intensity while -log10(Benjamini-Hochberg adjusted p-value) is represented by the bubble size. P-values were calculated by two-sided Wilcoxon-Test and only BH adjusted p-values <0.05 as reported.

Supplementary Figure 7

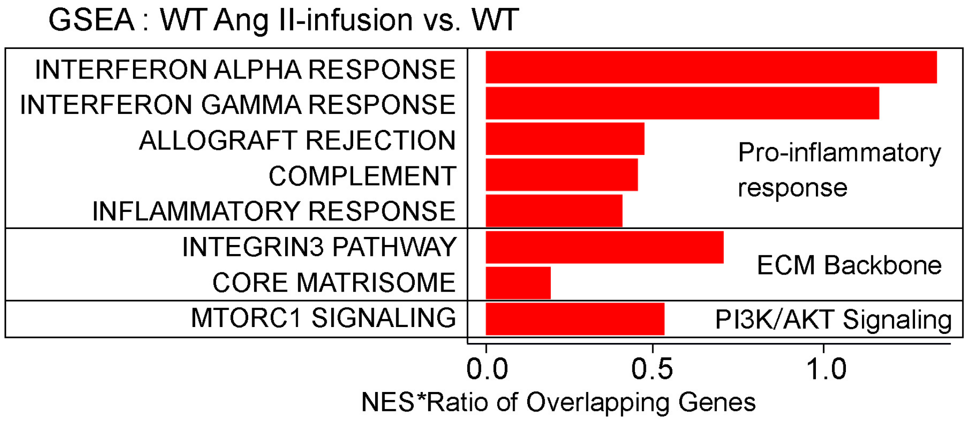

Supplementary Figure 7. Activation of pathways in cardiac macrophages at day 7 following Ang II infusion. ScRNA-seq gene set enrichment analysis (GSEA) of differentially expressed genes (DEGs) between WT cardiac macrophages following Ang-II infusion (500ng/kg/min, 7 days). Pathways are grouped in 3 meta-pathways: “Pro-inflammatory response”, “Extracellular Matrix (ECM) Backbone and “PI3K/AKT Signaling”. Scores for each pathway are calculated as the product of the GSEA normalized enrichment score (NES) and the ratio of overlapping genes with the entire canonical gene set (see Methods for details).

Supplementary Figure 8

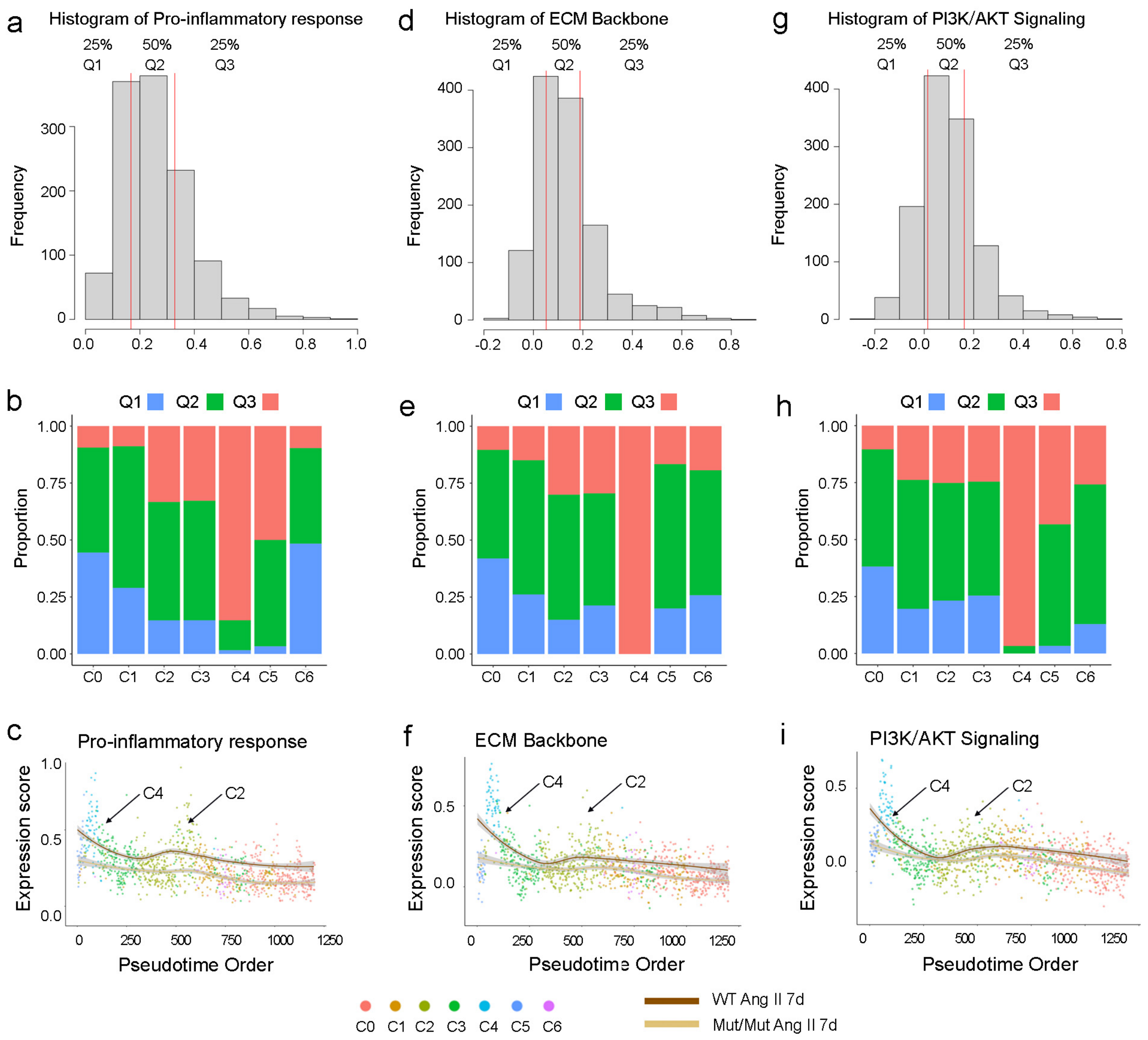

Supplementary Figure 8. Deconvolution of WWP2-regulated pro-inflammatory and pro-fibrotic response pathways in cardiac macrophage subclusters. a. Average expression score of the GSEA-derived upregulated pro-inflammatory response pathway among macrophage subclusters. Each subcluster is represented as three quartiles: Q1, low 25% in expression scores; Q2, middle 50% in expression scores; and Q3, 75% in expression scores. **b.** Distribution of the three quartiles for GSEA-derived pro-inflammatory response pathway among cardiac macrophage subclusters. **c.** Average expression score of pro-inflammatory response pathway plotted against the monocle-derived pseudotime for macrophage subclusters identified after treatment with Ang-II infusion (500ng/kg/min, 7 days) in WWP2$^{Mut/Mut}$ and WT mice. Similar analyses for the ECM Backbone (**d-f**) and the PI3K/AKT signaling (**g-i**) are reported.

Supplementary Figure 9

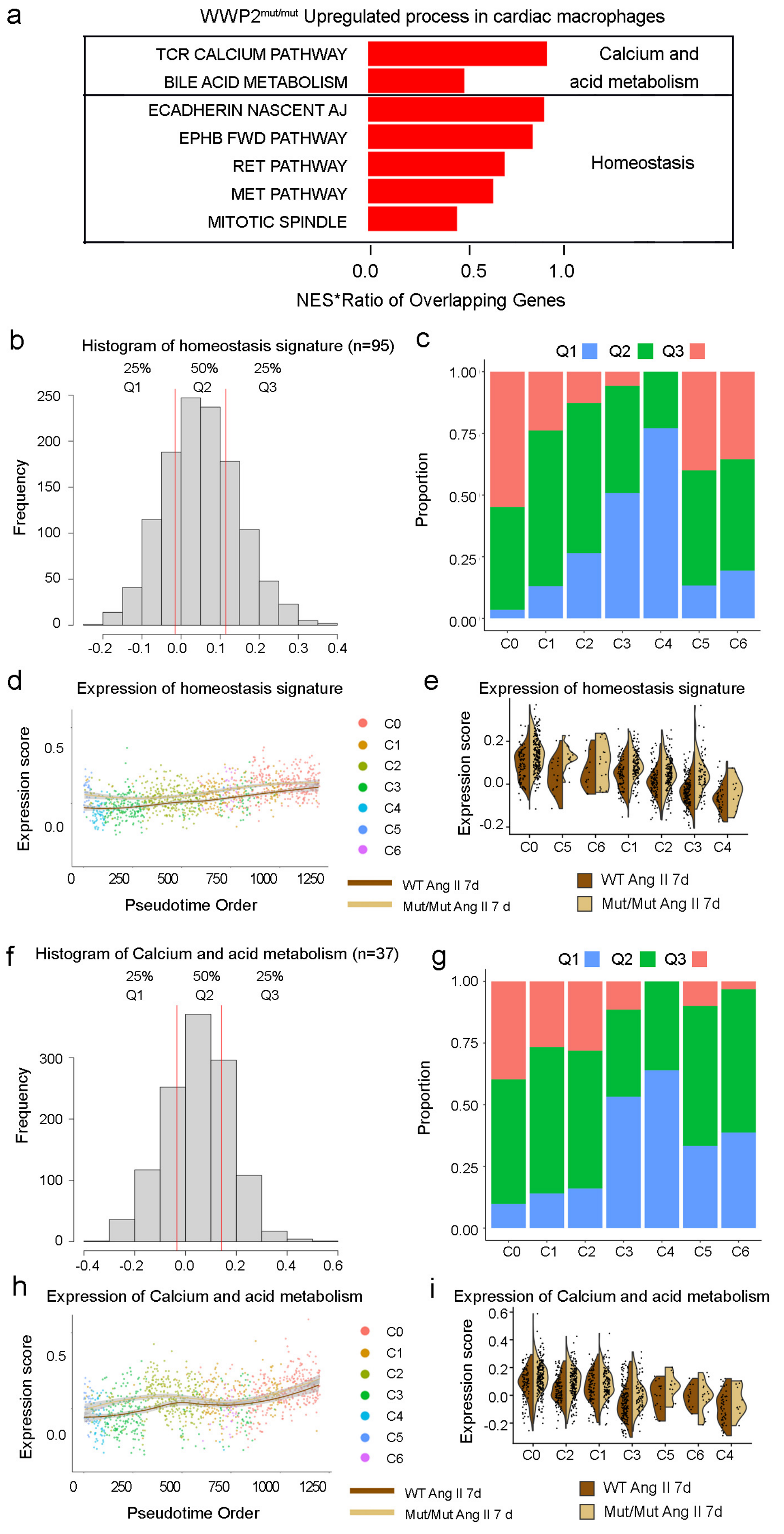

Supplementary Figure 9. ScRNA-seq analysis of up-regulated pathways in cardiac macrophages in WWP2$^{Mut/Mut}$ mice. a. Top up-regulated pathways from gene set enrichment analysis (GSEA) of differentially expressed genes between Ang II-treated (500ng/kg/min, 7 days) WWP2$^{Mut/Mut}$ and WT cardiac macrophages. Pathways are grouped into “Homeostasis” and “Calcium and Acid Metabolism” meta-pathways. Scores for each pathway are calculated as the product of the normalized enrichment score (NES) and the ratio of overlapping genes with the entire canonical gene set. Average expression score (**b**) and distribution (**c**) of ‘homeostasis GSEA pathway’ among macrophage subclusters. Average expression score of ‘homeostasis GSEA pathway’ plotted against monocle-derived pseudotime (**d**), and macrophage subclusters (**e**) for each macrophage subcluster identified by scRNA-seq in LV from WWP2$^{Mut/Mut}$ and WT mice treated with Ang-II infusion (500ng/kg/min, 7 days). Similar data for “calcium and acid metabolism” GSEA pathway are shown in panels **f-i**.

Supplementary Figure 10

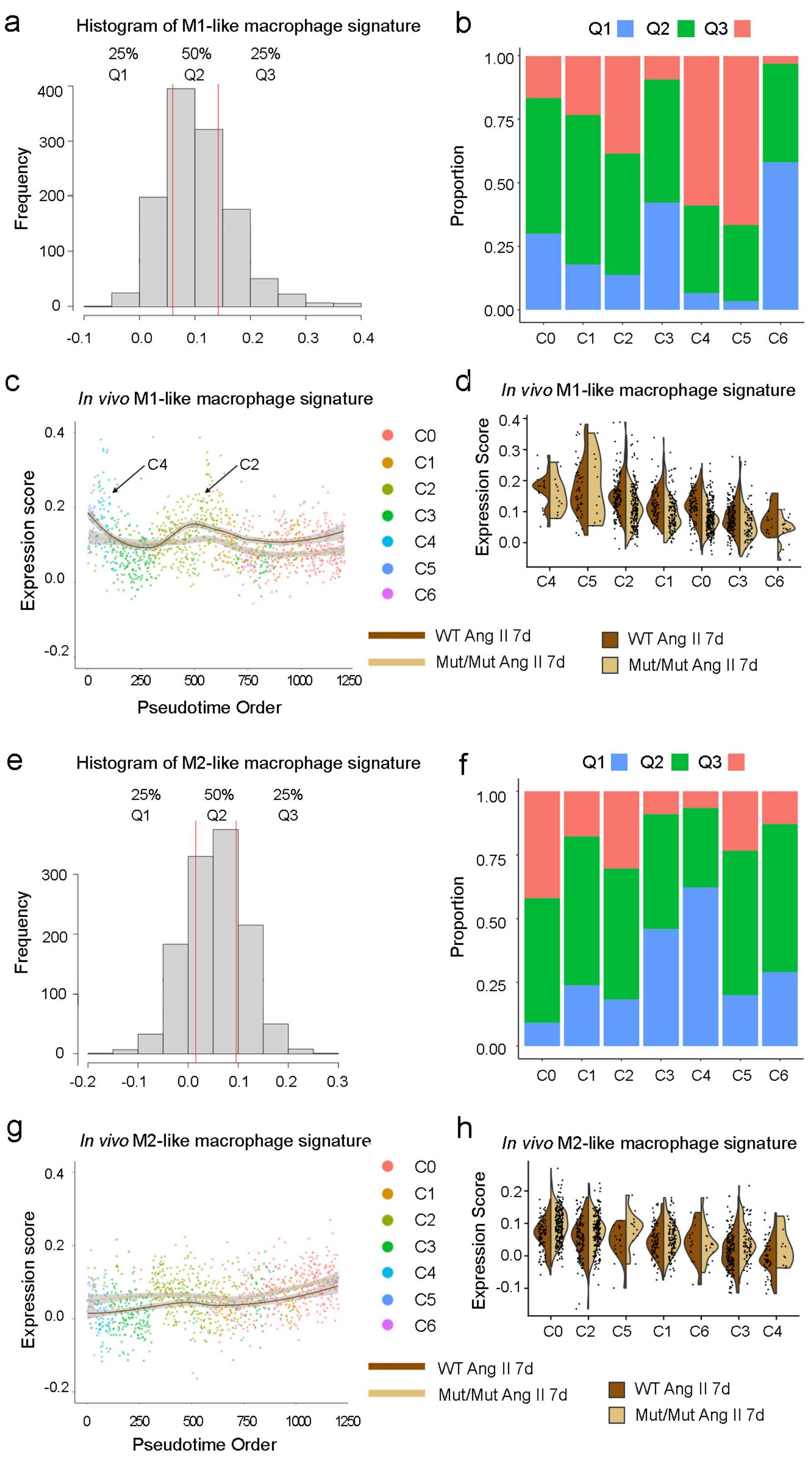

Supplementary Figure 10. WWP2 mutation shifts macrophage polarization from M1-like to M2-like. ScRNA-seq analysis of average expression score (**a**) and distribution (**b**) of *in* vivo M1-like transcriptomic signature (defined as described in [5]) among the cardiac macrophage subclusters. Average expression score of M1-like transcriptomic signature plotted against

monocle-derived pseudotime (**c**) and macrophage subclusters (**d**) in cardiac macrophages derived from $WWP2^{Mut/Mut}$ and WT mice after treatment with Ang-II infusion (500ng/kg/min, 7 days). Similar data for the *in vivo* M2-like transcriptomic signature (defined as described in [5]) are shown in panels **e-h**.

Supplementary Figure 11

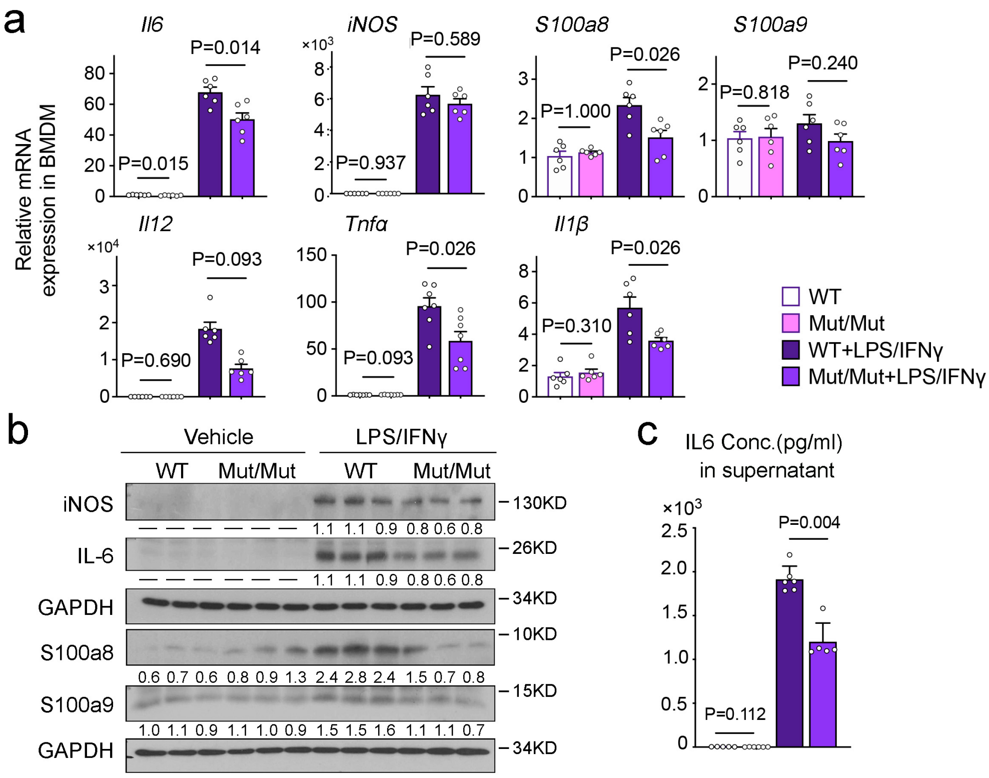

Supplementary Figure 11. Regulation of 'M1-like' genes by WWP2 in bone marrow derived macrophages (BMDM). a. Relative mRNA expression of pro-inflammatory genes in BMDMs from WT and WWP2$^{Mut/Mut}$ mice with or without LPS/IFNγ stimuli (LPS, 100ng/ml and IFNγ, 10ng/ml; 4 hrs). **b.** Representative western blot in BMDMs from WT and WWP2$^{Mut/Mut}$ mice showing the protein levels of iNOS, IL-6, S100a8 and S100a9 with or without LPS/IFNγ stimuli (LPS, 100ng/ml and IFNγ, 10ng/ml; 4 hrs). **c.** Secreted IL6 levels measured in supernatants from WT and WWP2$^{Mut/Mut}$ BMDMs with or without LPS/IFNγ (LPS, 100ng/ml and IFNγ, 10ng/ml; 4 hrs). Statistical significance calculated by non-parametric Mann-Whitney U test, n=5-6 for each group, data are shown as dot-plots with mean ± SD.

Supplementary Figure 12

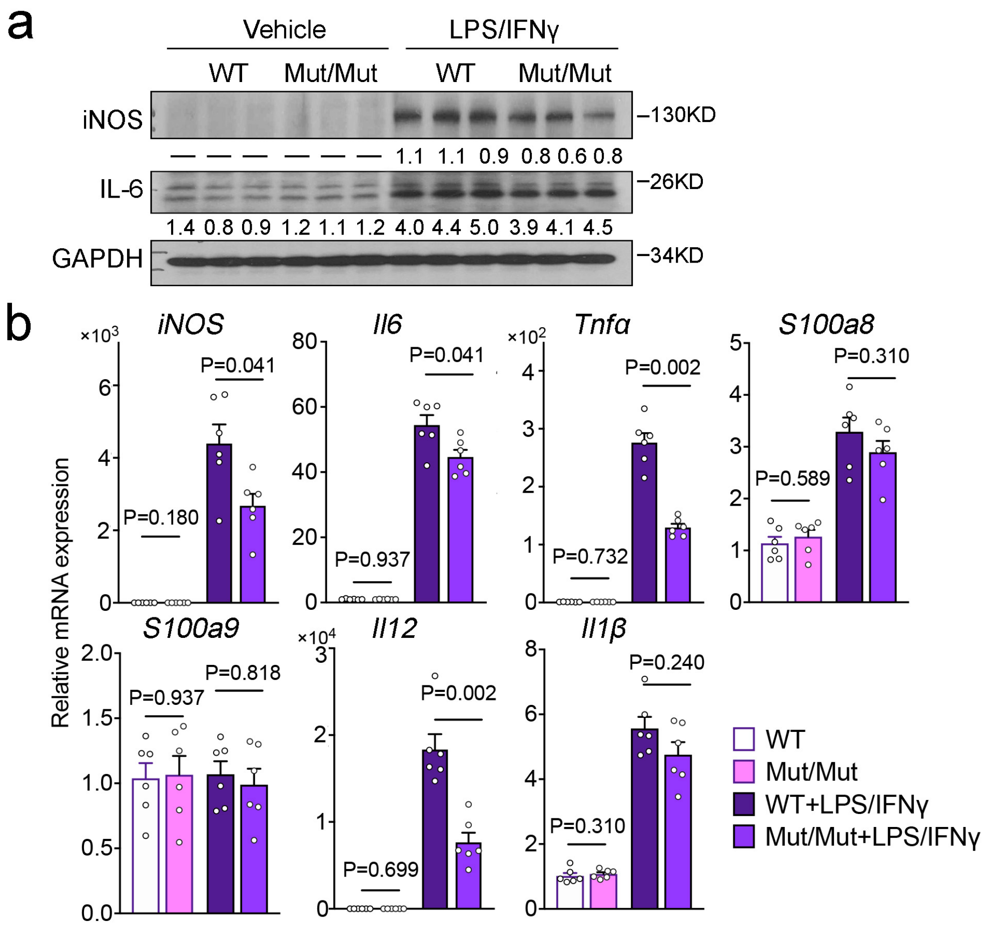

Supplementary Figure 12. Regulation of 'M1-like' genes by WWP2 in spleen-derived macrophages (SDM). a. Representative western blots in SDMs from WT and WWP2$^{Mut/Mut}$ mice showing the protein levels of iNOS and IL-6 with or without LPS/IFNγ (LPS, 100ng/ml and IFNγ, 10ng/ml; 4 hrs). **b.** Relative mRNA expression of representative pro-inflammatory genes in SDMs from WT and WWP2$^{Mut/Mut}$ mice with or without LPS/IFNγ (LPS, 100ng/ml and IFNγ, 10ng/ml; 4 hrs). Statistical significance calculated by non-parametric Mann-Whitney U test, n=5-6 for each group, data are shown as dot-plots with mean ± SD.

Supplementary Figure 13

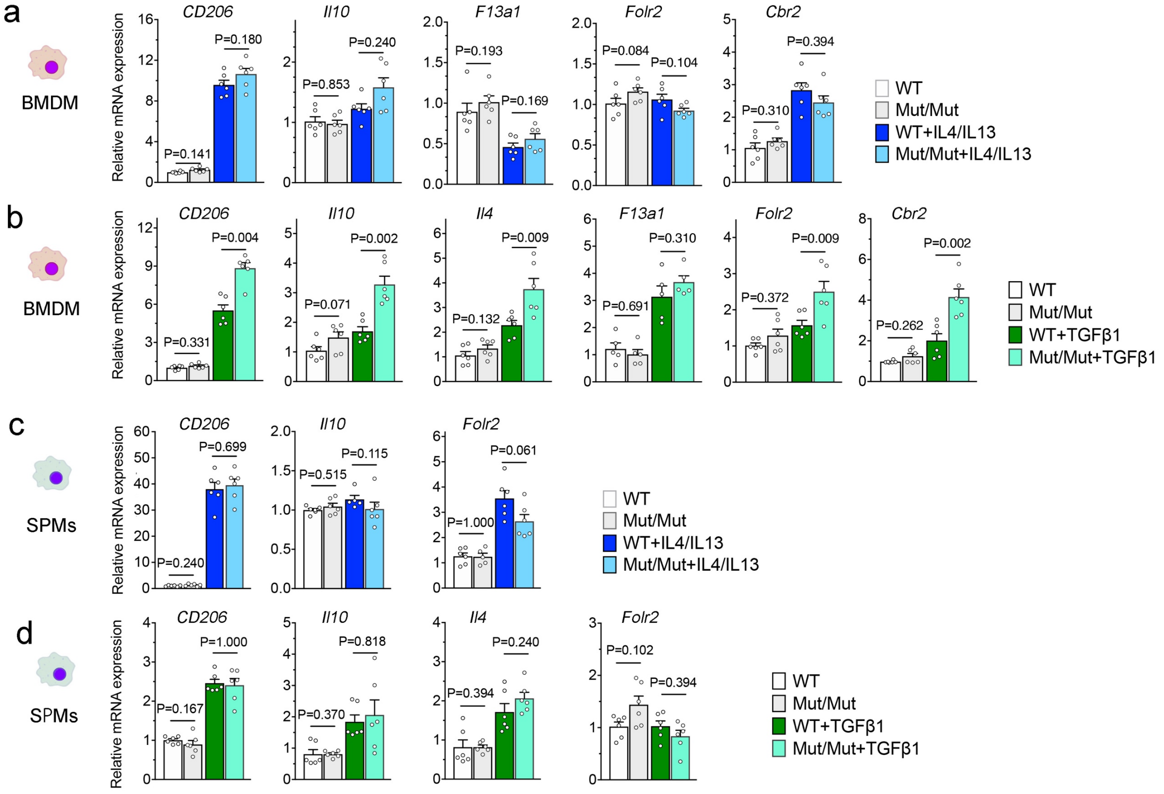

Supplementary Figure 13. Regulation of 'M2-like' genes by WWP2 in BMDMs and SDMs. Relative *in vitro* mRNA expression of representative 'M2-like' genes in macrophages isolated from WT and WWP2$^{Mut/Mut}$ mice. Gene expression is shown for BMDMs with or without IL4/IL13 (**a**) and TGF**β**1 (**b**) stimuli, and for SDMs with or without IL4/IL13 (**c**) and TGF**β**1 (**d**) stimuli. IL4 /IL13: IL4 (10ng/ml) + IL13 (10ng/ml), 8 hrs; TGF**β**1: 5ng/ml 24 hrs. Statistical significance calculated by non-parametric Mann-Whitney U test, n=5-6 for each group; data are shown as dot-plots with mean ± SD.

Supplementary Figure 14

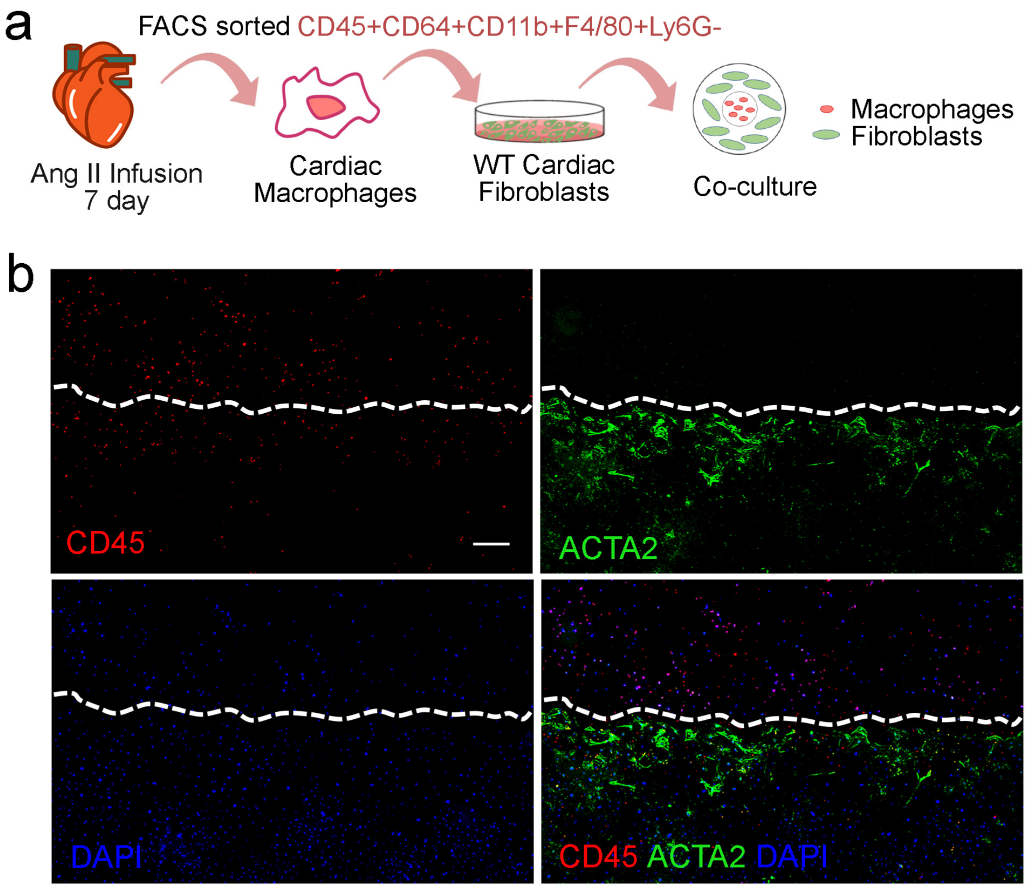

Supplementary Figure 14. Cardiac macrophage and fibroblast co-culture *ex vivo*. a. Schematic of macrophage-(myo)fibroblast co-culture experimental setup *ex vivo*. **b.** Representative immunofluorescence images of cardiac macrophages (anti-CD45, in red) and fibroblasts (anti-ACTA2, in green) after 72 hrs of co-culture. Cardiac macrophages were sorted from the heart (left ventricle) post-7 days following Ang II-infusion (500ng/kg/min), and primary fibroblasts were cultured from WT mouse hearts (left ventricle) and used at early passage (P2).

Supplementary Figure 15

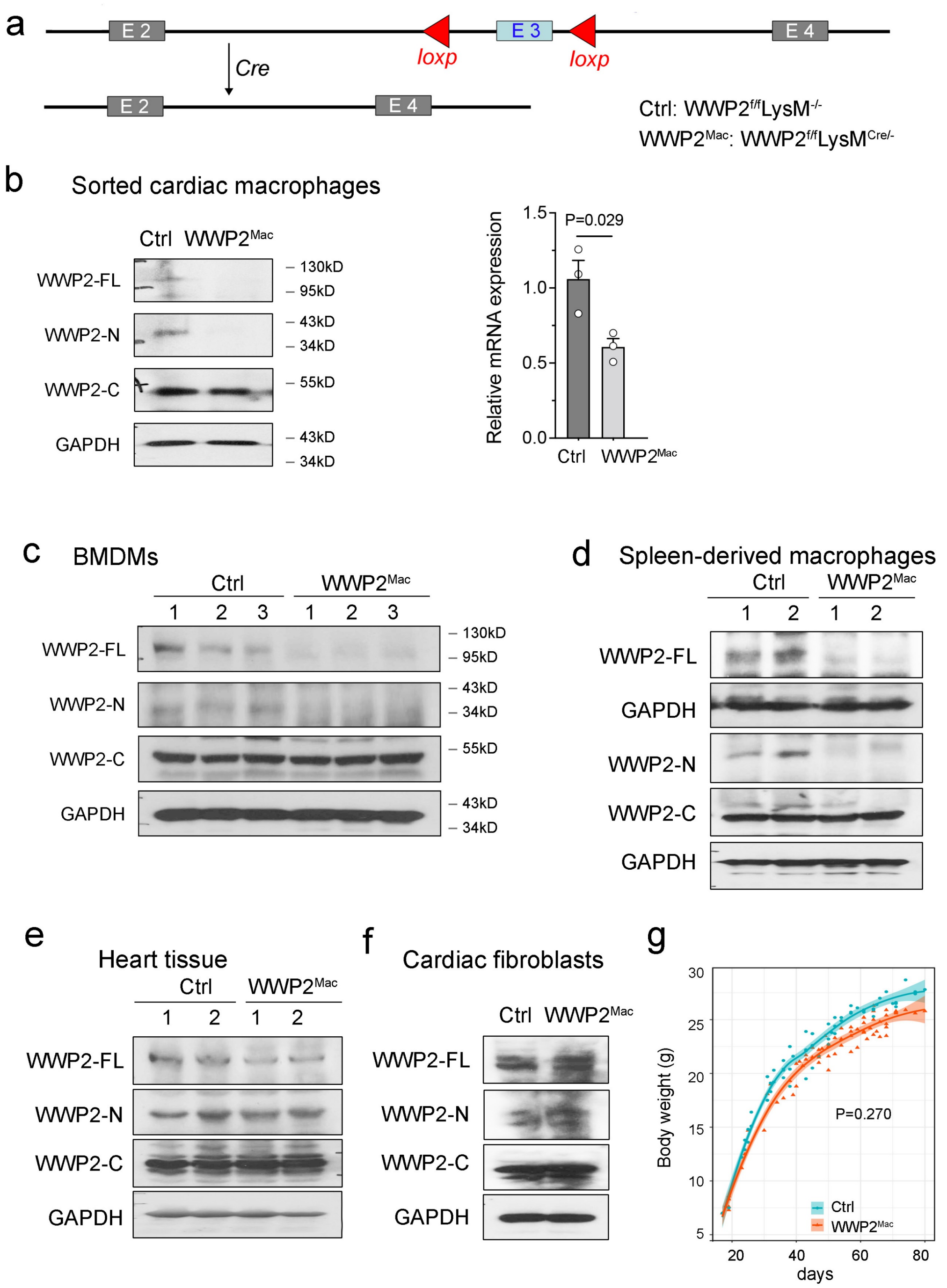

Supplementary Figure 15. Generation and phenotyping of WWP2$^{flox/flox}$LysM^{cre} mice. a. Schematic representation of *Wwp2* gene targeting to generate mice with simultaneous insertion of loxP sites bracketing exon 3. **b.** Representative WWP2 western blot (*left*, WWP2-FL: full-length isoform, WWP2-N: N-terminal isoform and WWP2-C: C-terminal isoform, as previously detailed in [6]) and relative *Wwp2* mRNA levels (*right,* with primers targeting exon 9 of the WWP2-FL) in cardiac macrophages isolated from Ctrl and WWP2^{Mac} mice. n=3 per experimental group, statistical significance calculated by non-parametric Mann-Whitney U test; data are shown as dot-plots with mean ± SD. **c-d.** Representative western blot images of WWP2 protein levels in BMDMs (**c**) and spleen-derived macrophages (SDMs) (**d**). **e-f.** Western blot of WWP2 isoforms in left ventricle tissue (**e**) and in primary cultured cardiac (myo)fibroblasts (**f**) derived from WWP2$^{flox/flox}$LysM$^{-/-}$ and WWP2$^{flox/flox}$LysM^{cre} mice. **g.** Body

weight changes in male Ctrl and $WWP2^{Mac}$ mice during a period from 20 to 80 days. n=4-8 for each experimental group; statistical significance was assessed by repeated-measures *t*-test (two-tailed). Description of experimental groups: $WWP2^{flox/flox}LysM^{-/-}$ is indicated in short as $WWP2^{f/f}LysM^{-/-}$ and used as control (Ctrl), and $WWP2^{flox/flox}LysM^{cre}$ is indicated in short as $WWP2^{f/f}LysM^{cre}$ and refers to the myeloid specific WWP2 knock-out mice ($WWP2^{Mac}$).

Supplementary Figure 16

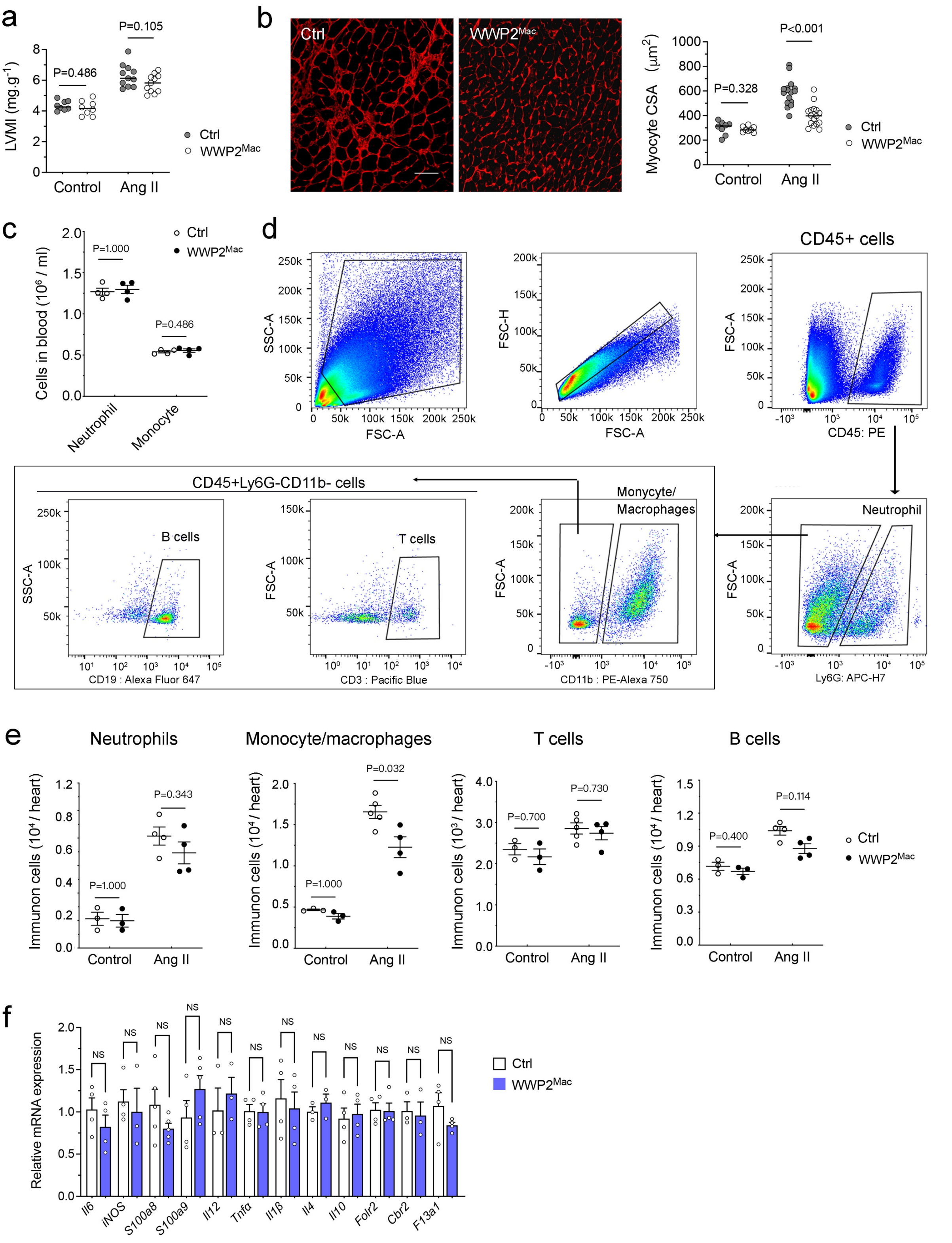

Supplementary Figure 16. Cardiac hypertrophy and details on circulating immune cells in WWP2^{Mac} mice. a. Left ventricular mass index (LVMI, mg/g) was compared between Ctrl and WWP2^{Mac} mice before and after Ang II-infusion (500ng/kg/min, 28 days). **b.** WGA staining (red) of Ang II infused hearts (LV) shows hypertrophic myocytes (*left*) with relatively higher mean Cardiomyocyte Cross-Sectional Area (CSA), (*right*, n=8-16, statistical significance

assessed by non-parametric Mann-Whitney U test, data are shown as dot-plots with mean ± SD). Scale bar: 50 µm. **c.** Quantification of peripheral neutrophils and monocytes (number/ml) in Ctrl and $WWP2^{Mac}$ mice after Ang II infusion (500ng/kg/min, 7 days). n=4-5 per experimental group; statistical significance calculated by non-parametric Mann-Whitney U test; data are shown as dot-plots with mean ± SD. **d.** Representative flow cytometry plots of the gating used to characterize cardiac neutrophils, monocyte/macrophages, T cells and B cells (Live/$CD45^{+}Ly6G^{+}$, live/CD45+ $CD11b^{+}$ $Ly6C^{+}$ $Ly6G^{-}$, live/CD45+ $CD11b^{-}$ $Ly6C^{-}$ $CD3^{+}$ and live/CD45+ $CD11b^{-}$ $Ly6C^{-}$ $CD19^{+}$). **e.** Quantification of cardiac neutrophils, monocyte/macrophages, T cells and B cells in Ctrl and $WWP2^{Mac}$ mice with or without Ang II infusion (500ng/kg/min, 7 days). n=3-5 for each experimental group; statistical significance calculated by non-parametric Mann-Whitney U test; data are shown as dot-plots with mean ± SD. **f.** qRT-PCR measuring mRNA expression of selected pro-inflammatory and homeostatic/repair genes in macrophages sorted from Ctrl and $WWP2^{Mac}$ mice heart (left ventricle) without Ang-II infusion. n=5-8 for each experimental group; statistical significance calculated by non-parametric Mann-Whitney U test; data are shown as dot-plots with mean ± SD.

Supplementary Figure 17

a

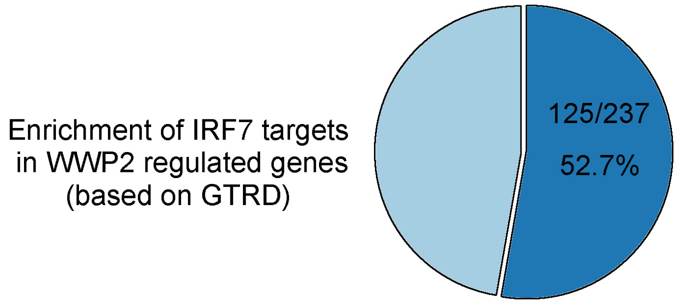

b

IRF7-*Ccl5*	P1-B1	P2-B2	P3-B3
Location (to TSS)	-1583/ -1450	-202/ -36	2503/ 2732 (intron 2)
IRF7-*Irf7*	P1-B1	P2-B2/B3	P3-B4
Location (to TSS)	588/ 765(intron 2)	304/ 484 (intron 1)	-1326/ -1085

Supplementary Figure 17. WWP2 regulates the IRF7 regulon in cardiac macrophages. a. Breakdown of IRF7 targets enrichment in WWP2 regulated genes (237 transcripts), of which 125 (52.7%) are targets of IRF7, based on the Gene Transcription Regulation Database (GTRD), https://gtrd.biouml.org/. **b.** Details of primers and location (bp) that were used in the ChIP-qPCR analysis of *Ccl5* and *Irf7* loci. The location of *Ccl5* and *Irf7* PCR products and co-localizing IRF7 binding site motifs are shown in Figure 5g.

Supplementary Figure 18

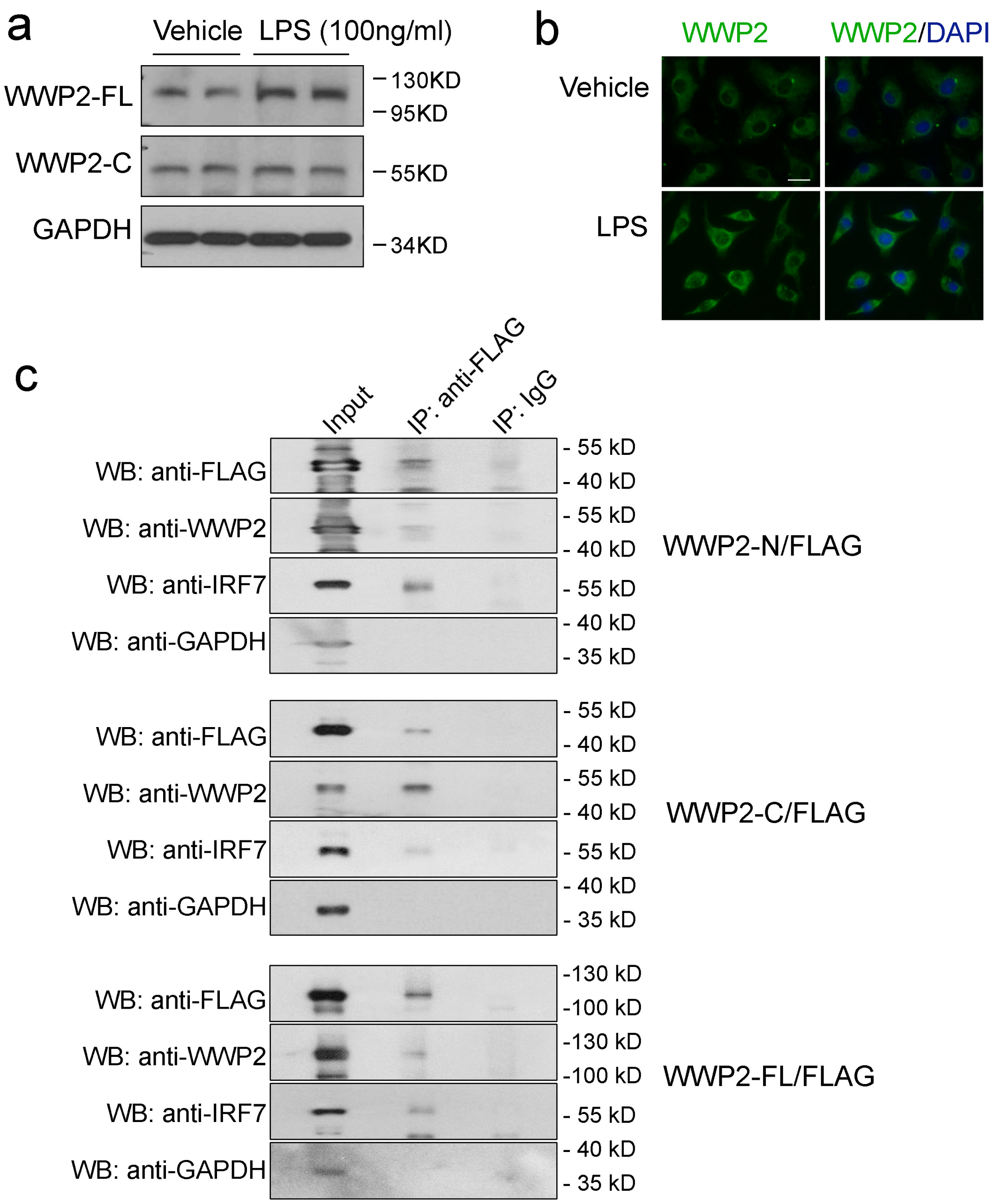

Supplementary Figure 18. Expression of WWP2 in basal and activated BMDMs following LPS treatment. a. Representative western blot of WWP2 isoforms (WWP2-FL and WWP2-C, detailed in Supplementary Figure 15) in BMDMs after LPS treatment (100ng/ml, 4 hrs). **b.** Immunofluorescence analysis of WWP2 (in green) showing cytoplasmic localization with or without LPS treatment (100ng/ml, 4 hrs) in WT BMDMs. Scale bar: 20 µm. **c.** Hek293 cells were transfected with WWP2-Flag isoforms and immunoprecipitated with anti-FLAG antibodies or IgG controls, followed by western blotting probed with antibodies as indicated.

Supplementary Figure 19

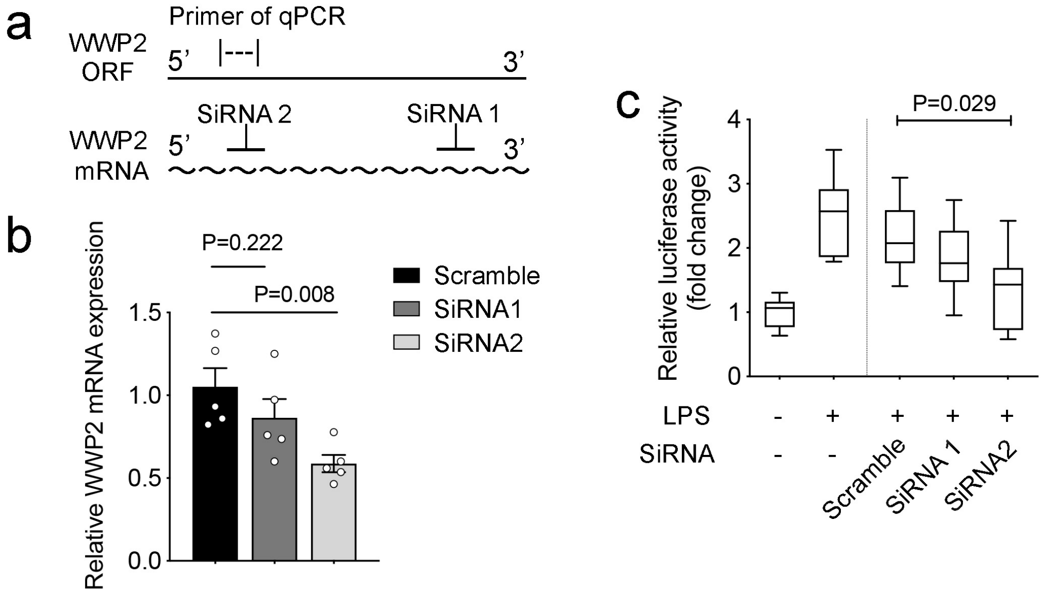

Supplementary Figure 19. WWP2 regulates IRF7 Interferon-Sensitive Response Element (ISRE) activity. a. Schematics of the genomic location of the primer-pair and two siRNA sequences targeting *Wwp2* mRNA. **b-c.** Bar-dot plot showing relative *Wwp2* mRNA expression (**b**) and box-and-whisker plot showing ISRE luciferase activity (**c**) in NIH-3T3 cells co-treated with LPS (100ng/ml, 4 hrs) and siRNA targeting *Wwp2.* Statistical significance calculated by non-parametric Mann-Whitney U test; n=4-5 for each experimental group.

Supplementary Figure 20

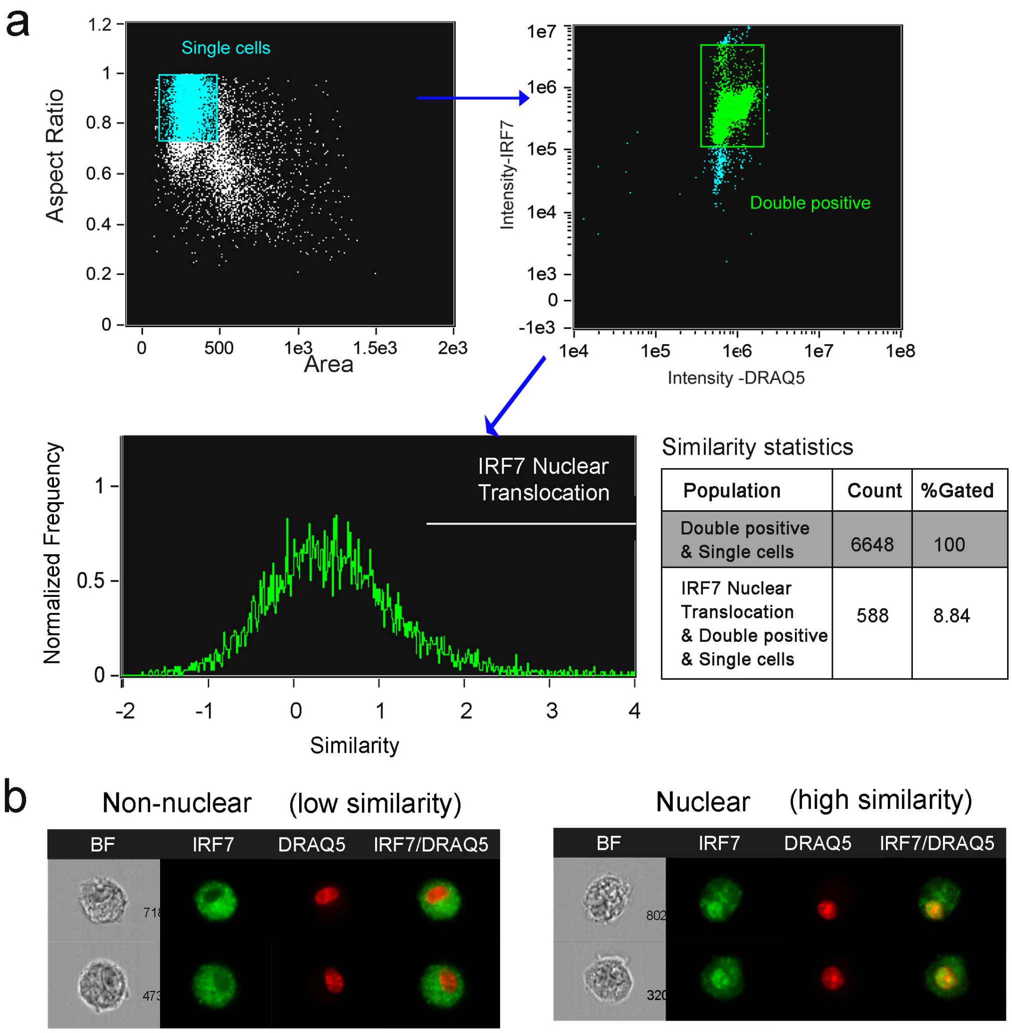

Supplementary Figure 20. IRF7 nuclear translocation in BMDMs. a. Representative gating strategy for the quantification of IRF7 nuclear translocation by flow cytometry imaging. A similarity score of ≥1.5 for IRF7 and DRAQ5 is used to define cells with nuclear IRF7. **b.** Representative images of in-cell IRF7 and p-IRF7 from 10,000 acquired events by imaging flow cytometry showing distinct non-nuclear (left) and nuclear (right) distribution patterns.

Supplementary Table 1. Primers for quantitative reverse transcription PCR detection.

Gene	Forward (5'-3')	Reverse (3'-5')
Wwp2-P1	TTTGAGAAGTCCCAGCTTACCC	CTCCAGACCTTCAGATCCAAATG
Wwp2-P2	CAGCGCCCAACTGCTGAGTA	TGGTAGAGGAATCTTTGGCTG A
Wwp2-P3	ACCAAGAGCAGCAAGCAGAT	GTCTCCTCGATGGCGTACAG
18S rRNA	GTAACCCGTTGAACCCCATT	CCATCCAATCGGTAGTAGCG
Acta2	GTCCCAGACATCAGGGAGTAA	TCGGATACTTCAGCGTCAGGA
Col1a1	GCTCCTCTTAGGGGCCACT	CCACGTCTCACCATTGGGG
POSTN	CCTGCCCTTATATGCTCTGCT	AAACATGGTCAATAGGCATCACT
CTGF	CCTGCCCTTATATGCTCTGCT	AAACATGGTCAATAGGCATCACT
FN-EDA	CCCTAAAGGACTGGCATTCA	TCTGCAGTGTCGTCTTCACC
CCL2	TTAAAAACCTGGATCGGAACCAA	GCATTAGCTTCAGATTTACGGGT
CCL5	GCTGCTTTGCCTACCTCTCC	TCGAGTGACAAACACGACTGC
CCL7	TACCATGAGGTCACTTCAGATGC	GCACTCTCGGCCTACATTGG
CCL12	ATTTCCACACTTCTATGCCTCCT	ATCCAGTATGGTCCTGAAGATCA
CCL22	AGGTCCCTATGGTGCCAATGT	CGGCAGGATTTTGAGGTCCA
CCL24	ATTCTGTGACCATCCCCTCAT	TGTATGTGCCTCTGAACCCAC
IL1β	GCAACTGTTCCTGAACTCAACT	ATCTTTTGGGGTCCGTCAACT
IL4	GGTCTCAACCCCCAGCTAGT	GCCGATGATCTCTCTCAAGTGAT
IL6	TAGTCCTTCCTACCCCAATTTCC	TTGGTCCTTAGCCACTCCTTC
IL10	GCTCTTACTGACTGGCATGAG	CGCAGCTCTAGGAGCATGTG
IL12	TGGTTTGCCATCGTTTTGCTG	ACAGGTGAGGTTCACTGTTTCT
iNOS	GTTCTCAGCCCAACAATACAAGA	GTGGACGGGTCGATGTCAC
S100a8	AAATCACCATGCCCTCTACAAG	CCCACTTTTATCACCATCGCAA
S100a9	CCTTCTCAGATGGAGCGCAG	TGTCCAGGTCCTCCATGATG
TGFβ1	CTCCCGTGGCTTCTAGTGC	GCCTTAGTTTGGACAGGATCTG
Arg1	CTCCAAGCCAAAGTCCTTAGAG	AGGAGCTGTCATTAGGGACATC
CD206	CTCTGTTCAGCTATTGGACGC	CGGAATTTCTGGGATTCAGCTTC
Folr2	GGGACAGAACAGACCTACTCA	AGTCAGCCTTGTGTAGCTCCT
Cbr2	GGGCAGGGAAAGGGATTGG	CCACACACACGGGCTCTATTC

F13a1	GAGCAGTCCCGCCCAATAAC	CCCTCTGCGGACAATCAACTTA
IRF7	GAGACTGGCTATTGGGGGAG	GACCGAAATGCTTCCAGGG
IFNα	GGATGTGACCTTCCTCAGACTC	ACCTTCTCCTGCGGGAATCCAA
IFNβ	CAGCTCCAAGAAAGGACGAAC	GGCAGTGTAACTCTTCTGCAT
IFNγ	ATGAACGCTACACACTGCATC	CCATCCTTTTGCCAGTTCCTC
TNFα	CCCTCACACTCAGATCATCTTCT	GCTACGACGTGGGCTACAG
IRF7-CCL5 P1	CTTGGTGGGCACTCTGTCTG	ATGGTCCCAAGTCTGTCCTG
IRF7-CCL5 P2	CTGGACTGGAGGGCAGTTAG	AGCCAGGGTAGCAGAGGAAG
IRF7-CCL5 P3	TGGTGCACAAATACATGCAG	TGCCGGCTCATAATAGGTTC
IRF7-IRF7 P1	CCTAAAGGTCTACCCACTGCAATA	AGTCAAGGGTTGTGTCCATCCT
IRF7-IRF7 P2	GGTAGGCATGGAGACAGTGG	GGTGTTAATCCAGCGTCCGA
IRF7-IRF7 P2	CAAACCTCATCTGTGACCCTCA	CAGCTATTGCAAATAAGGCCCC

Supplementary references

1. Skelly, D.A., et al., *Single-Cell Transcriptional Profiling Reveals Cellular Diversity and Intercommunication in the Mouse Heart.* Cell Rep, 2018. **22**(3): p. 600-610.
2. Dick, S.A., et al., *Self-renewing resident cardiac macrophages limit adverse remodeling following myocardial infarction.* Nat Immunol, 2019. **20**(1): p. 29-39.
3. Zaman, R., et al., *Selective loss of resident macrophage-derived insulin-like growth factor-1 abolishes adaptive cardiac growth to stress.* Immunity, 2021. **54**(9): p. 2057-2071 e6.
4. Wong, N.R., et al., *Resident cardiac macrophages mediate adaptive myocardial remodeling.* Immunity, 2021.
5. Orecchioni, M., et al., *Macrophage Polarization: Different Gene Signatures in M1(LPS+) vs. Classically and M2(LPS-) vs. Alternatively Activated Macrophages.* Front Immunol, 2019. **10**: p. 1084.
6. Chen, H., et al., *WWP2 regulates pathological cardiac fibrosis by modulating SMAD2 signaling.* Nat Commun, 2019. **10**(1): p. 3616.
