# Supplementary material for: The E3 ubiquitin ligase WWP2 regulates pro-fibrogenic monocyte infiltration and activity in heart fibrosis
Source: Nat Commun. 2022 Nov 30;13:7375. doi: 10.1038/s41467-022-34971-6 (PMC9712659; doi:10.1038/s41467-022-34971-6)
Supplement: Supplementary file 3 — Reporting Summary [file 41467_2022_34971_MOESM3_ESM.pdf]

nature portfolio

Corresponding author(s): Huimei Chen, Jacques Behmoaras and Enrico Petretto

Last updated by author(s): Oct 28, 2022

Reporting Summary

Nature Portfolio wishes to improve the reproducibility of the work that we publish. This form provides structure for consistency and transparency in reporting. For further information on Nature Portfolio policies, see our Editorial Policies and the Editorial Policy Checklist.

Statistics

For all statistical analyses, confirm that the following items are present in the figure legend, table legend, main text, or Methods section.

n/a	Confirmed	
☐	☒	The exact sample size (*n*) for each experimental group/condition, given as a discrete number and unit of measurement
☐	☒	A statement on whether measurements were taken from distinct samples or whether the same sample was measured repeatedly
☐	☒	The statistical test(s) used AND whether they are one- or two-sided<br>*Only common tests should be described solely by name; describe more complex techniques in the Methods section.*
☐	☒	A description of all covariates tested
☐	☒	A description of any assumptions or corrections, such as tests of normality and adjustment for multiple comparisons
☐	☒	A full description of the statistical parameters including central tendency (e.g. means) or other basic estimates (e.g. regression coefficient) AND variation (e.g. standard deviation) or associated estimates of uncertainty (e.g. confidence intervals)
☐	☒	For null hypothesis testing, the test statistic (e.g. *F*, *t*, *r*) with confidence intervals, effect sizes, degrees of freedom and *P* value noted<br>*Give P values as exact values whenever suitable.*
☒	☐	For Bayesian analysis, information on the choice of priors and Markov chain Monte Carlo settings
☒	☐	For hierarchical and complex designs, identification of the appropriate level for tests and full reporting of outcomes
☒	☐	Estimates of effect sizes (e.g. Cohen's *d*, Pearson's *r*), indicating how they were calculated

Our web collection on statistics for biologists contains articles on many of the points above.

Software and code

Policy information about availability of computer code

Data collection	Isolated single cell suspensions were converted to barcoded scRNA-seq libraries by using the Chromium Single Cell 3'Library, Gel Bead & Multiplex Kit, and Chip Kit V3, loading an estimated 7,000–12,000 cells per library/well and following the manufacturer's instructions. Indexed libraries were sequenced using Illumina HiSeq 4000 sequencer, where 150bp pair-end sequences were obtained. Sequencing reads were aligned and quantified to the mouse genome GRCm38 (mm10-3.0.0 provided by 10x Genomics) using Cellranger count (v3.1.0).
Data analysis	Single cell RNA sequence data were analyzed by following analysis packages: Seurat(v4.1.0); readxl(v1.3.1); ggplot2(v3.3.5); scds (v1.6.0); scran(v1.18.7); RColorBrewer(v1.1.2); ggrepel(v0.9.1);pheatmap(v1.0.12); dplyr (v1.0.7); ggpubr (v0.4.0); reshape2(v1.4.4); tidyverse(v1.3.1); nichenetr(v1.0.0); VennDiagram(v1.7.1); magrittr(v2.0.2); clusterProfiler(v3.18.1); org.Mm.eg.db(v3.12.0); org.Hs.eg.db(v3.12.0); biomaRt(v2.46.3); Biobase(v2.50.0); stringr(v1.4.0); psych(v2.1.9); ggbeeswarm(v0.6.0); ggridges(v0.5.3); Matrix(v1.4.0); ggnewscale(v0.4.5); monocle3(v1.0.0); SeuratWrappers(v0.3.0); gridExtra(v2.3); data.table(v1.14.2); Binarize(v1.3); CellphoneDB(v2.0); pySCENIC (v0.11.2); plotly (v4.9.3);PharmacoGx(v2.2.4); Cellranger (v3.1.0); lsa(v0.73.2); AnnotationDbi(v1.52.0); CoreGx(v1.2.0); DOSE(v3.16.0); matrixStats(v0.61.0) The immunofluorescent data were analyzed by ImageJ 1.53k software, the Flow cytometry data were analyzed by FlowJo v10, and the ImageStream data were analyzed by IDEAS software package.

For manuscripts utilizing custom algorithms or software that are central to the research but not yet described in published literature, software must be made available to editors and reviewers. We strongly encourage code deposition in a community repository (e.g. GitHub). See the Nature Portfolio guidelines for submitting code & software for further information.

Data

Policy information about availability of data

All manuscripts must include a data availability statement. This statement should provide the following information, where applicable:

- Accession codes, unique identifiers, or web links for publicly available datasets
- A description of any restrictions on data availability
- For clinical datasets or third party data, please ensure that the statement adheres to our policy

All the data generated in this study supporting the main findings have been deposited to NCBI's Gene Expression Omnibus (GEO) and accessible through GEO Series accession number GSE198003 [https://www.ncbi.nlm.nih.gov/geo/query/acc.cgi?acc=GSE198003]. The processed data are comprised in Source data and provided as a Source Data file.

Field-specific reporting

Please select the one below that is the best fit for your research. If you are not sure, read the appropriate sections before making your selection.

☒ Life sciences ☐ Behavioural & social sciences ☐ Ecological, evolutionary & environmental sciences

For a reference copy of the document with all sections, see nature.com/documents/nr-reporting-summary-flat.pdf

Life sciences study design

All studies must disclose on these points even when the disclosure is negative.

Sample size	1. In the signal cell seq analysis, the sufficient cells were samples as suggested in navinlab.com/SCOPIT.<br>2. Sample size of mice groups was determined by power analysis based on the observed variability. In detail, 1) phenotype analysis, n>=8 per group between WT and MUT/MUT OR between WT and WWP2Mac were used . 2) molecular analysis, n>=3 WT and MUT/MUT OR between WT and WWP2Mac within same genetic background.<br>For in vivo studies phenotype, sample size calculations were based on previous data, suggesting that detection of a greater than 80% power at the 5% α-level would require a minimum of 8 animals per experimental group. For molecular analysis, including single cell sequence analysis, sample size was determined based on previously published studies and no powerl analysis was employed.
Data exclusions	No data was excluded from the analysis.
Replication	All experiments were replicated, and the number of replicates are stated in each figure legend.
Randomization	When comparing mice with different genotypes, littermate mice were assigned to the WT and Mut/Mut or WT and WWP2Mac groups according to the results of genotyping and mice with the same genotype were randomly assigned to the control, AngII infusion group using a simple random-sampling approach.
Blinding	The experimenters were blinded to the grouping information during data collection and analysis.

Reporting for specific materials, systems and methods

We require information from authors about some types of materials, experimental systems and methods used in many studies. Here, indicate whether each material, system or method listed is relevant to your study. If you are not sure if a list item applies to your research, read the appropriate section before selecting a response.

Materials & experimental systems

n/a	Involved in the study	
☐	☒	Antibodies
☐	☒	Eukaryotic cell lines
☒	☐	Palaeontology and archaeology
☐	☒	Animals and other organisms
☒	☐	Human research participants
☒	☐	Clinical data
☒	☐	Dual use research of concern

Methods

n/a	Involved in the study	
☒	☐	ChIP-seq
☐	☒	Flow cytometry
☒	☐	MRI-based neuroimaging

Antibodies

Antibodies used	Blotting of the membrane was performed using anti-WWP2 (#A302-936A, Bethyl Laboratories, WB-1:500, Rabbit anti-human/mouse, WB validated in 293T, HeLa and Jurkat cell lines), anti-ACTA2 (#A5228, Sigma-Aldrich, Mouse monoclonal anti-human/mouse et al., WB-1:10,000, WB validated in HS-68, RAT2, LA4 and C2C12 cell lines), anti-Vimentin (#ab45939, Abcam, Rabbit anti-human/rat/

mouse, WB-1:500, WB validated in HeLa, Jurkat, HEK293and Ramos cell lines), anti-Periostin (#NBP1-30042, Novus Bio, Rabbit anti-human/mouse et al., WB-1:500, WB validated in mice cardiac and skeletal muscle and rat lung), anti-Fibronectin (#SAB4500974, Sigma-Aldrich, Rabbit anti human/mouse, WB-1:500, WB validated in HeLa cells), anti-Ubiquitin (#3933, CST, Rabbit anti human/mouse, WB- 1:500, WB validated in HeLa and NIH3T3 cell lines), IRF7 (#GTX01065, GeneTex, Rabbit anti-mouse/human, WB-1:500, WB validated without detail information), pIRF7(#24129S, CST, Rabbit anti Mouse Specific, WB-1:500, WB validated in HEK293T and RAW 264.7 cell lines), pIRF7 (#PA564834, Thermo fisher, Rabbit anti houman/mouse/Rat, WB-1:500, WB validated in LOVO whole cell lysates), S100A8 (#ab92331, Abcam, Rabbit anti mouse/human/Rat, WB- 1:500, validated in THP1 cell extract), S100A9 (#ab105472, Abcam, Rat anti mouse/human, WB-1:500, IF validated in FFPE mouse lung tissue), IFNß (#ab218229, Abcam, Rabbit anti mouse, WB-1:500, WB validated in RAW 264.7 mouse macrophage cell lines), IFNγ (#ab133566, Abcam, Rabbit anti human/mouse, WB-1:500, WB validated in MOLT4, Jurkat cell lines and Human kidney and lymph node lysates), CCL-2 (#ab25124, Abcam, , Rabbit anti mouse/Rat, WB-1:500, WB validated in Rat Spinal cord lysate), CCL-5 (sc-365826, SantaCruz, Mouse anti mouse/rat/hums, WB-1:500, WB validated in human recombinant fusion protein), iNOS (#ab15323, Abcam, Rabbit anti mouse/rat, WB-1:500, WB validated in mouse tissue and RAW 264.7 cell lines), IL-6 (#ab208113, Abcam, Rabbit anti mouse, WB-1:500, WB validated in recombinant mouse protein), Anti-FLAG (#F7425, Sigma-Aldrich, Rabbit, WB-1:1000, validated in HEK293T cells after transfer onto a nitrocellulose membrane at 100 V for 1h), Anti-Tubulin (#T5168, Sigma-Aldrich, Mouse anti human/mouse/etc., WB-1:5000, validated in HeLa, Jurkat, RAT2, NIH3T3, CHO, COS7 cell lines) and anti-GAPDH (#ab8245, Abcam, Mouse anti human/mouse/rat, WB-1:5000, validated in mouse and rat hippocampus whole cell lysates, Raji lymphoma cell line, HeLa, Jurkat, A431, HEK293 cell lines), Anti-Lamin A/C (#ab8984, Abcam, Mouse anti human/pig, WB-1:5000, WB validated in HT1080 cells). Blots were visualized with secondary antibodies including anti-Rabbit HRP (#A120-101P, Bethyl Laboratories, Rabbit, WB- 1:5000), anti- Mouse HRP (Bethyl laboratories, #A90-116P, Mouse, WB-1:5000), anti- Rat IgG whole molecule (#A9037, Sigma- Aldrich), Protein A- HRP (#101023, Thermofisher,1:1000).
Antibodies used For immunofluorescent staining were listed as following: anti-WWP2 (#A302-936A, Bethyl Laboratories, Rabbit, IF-1:100, IF validation information is not described), anti-CD68 (#ab125212, abcam, Rabbit anti mouse/rat, IF- 1:100, IF validated in mouse and rat liver tissues), anti Ki-67 (#ab15580, abcam, Rabbit anti mouse/human, IF-1:100, IF validated in HeLa cell, Rabbit retina cell, HAP1cells, mouse trachea and SK-N-SH cells), anti-ACTA2 (#A5228, Sigma-Aldrich, Mouse monoclonal anti-human/mouse et al., IF-1:100, IF validated in Rat2 cell line), anti-CD45 (#12-0451-83, invitrogen, rat anti mouse, IF-1:100, IF validation information is not described). Images were visualised using second antibodies, including Goat anti-Rabbit IgG (H+L) Cross-Adsorbed Secondary Antibody 488 (A11008, Thermo fisher, Goat, IF-1:500), Goat anti-Mouse IgG (H+L) Cross-Adsorbed Secondary Antibody 488 (A11001, Thermo fisher, Goat, IF-1:500), Goat anti-Rabbit IgG (H+L) Cross-Adsorbed Secondary Antibody 568 (A11011, Thermo fisher, Goat, IF-1:500), Goat anti-Mouse IgG (H+L) Cross-Adsorbed Secondary Antibody 568 (A11004, Thermo fisher, Goat, IF-1:500) and Goat anti-Rat IgG (H+L) Cross-Adsorbed Secondary Antibody 568 (A11077, Thermo fisher, Goat, IF-1:500). DAPI (#D1306, Thermo fisher) and Alexa Fluor 647 Conjugate Wheat Germ Agglutinin dye (#W32466, Life technologies holdings, IF- 1:750) were used to visualise the nuclei and membrane of myocytes.
Chromatin immunoprecipitation (ChIP) assay was performed with anti-IRF7 antibody (ABF 130, Merck, Rabbit anti mouse, WB and CHIP suggested for application). and anti-WWP2 (#A302-936A, Bethyl Laboratories, Rabbit anti mouse/human, WB-1:500, validated in 293T, CHIP validated in HeLa and Jurkat cell lines).
The detail antibodies used for flow-cytometry as following: Anti-Ly-6C Monoclonal Antibody PerCP-Cyanine5.5 (Rat anti mouse, #45-5932-82,Thermofisher, FACS-1:25, validated in mouse spleen), APC Rat anti mouse Anti-CD11b (#561690,BD Biosciences, FACS-1:100, validated in bone marrow myeloid cells), FITC Mouse Anti-Mouse CD45.2 (#561874, BD Biosciences, FACS-1:100, validated in mouse spleen), APC-H7 Rat Anti-Mouse Ly-6G (#565369, BD Biosciences, FACS-1:200, validated in mouse bone marrow leucocytes), PE Mouse anti-Mouse CD64 (#558455,BD Biosciences, FACS-1:100, validated in mouse bone marrow cells), BV421 Rat Anti-Mouse F4/80 (Rat,#565411, BD Biosciences FACS-1:100, validated in mouse spleen leucocytes), mouse anti-mouse CD45 (#561874, BD Biosciences, Mouse, FACS- 1:100, validated in mouse spleen), rat anti-mouse IRF7-PE (#12-5829-82, Bio-legend, Rat, FACS-1:100, validated in mouse Spleen). DRAQ5 (#564903, BD Pharmingen) was used as nuclear dye.

Validation

Full and unprocessed scanned images of the blots are shown in Supplementary figures. Validation information from the website of the antibody companies is listed in the detail of antibodies.

Eukaryotic cell lines

Policy information about cell lines

Cell line source(s)	NIH-3T3 from ATCC
Authentication	Not be authenticated and directly got from ATCC.
Mycoplasma contamination	The cells were not tested for mycoplasma contamination.
Commonly misidentified lines (See ICLAC register)	N/A

Animals and other organisms

Policy information about studies involving animals; ARRIVE guidelines recommended for reporting animal research

Laboratory animals

WWP2 mut/wt mice were generated based on C57BL/6J in the laboratory of Dr. Weiping Yu at Agency for Science, Technology and Research (A*STAR). The clone is kept in the vivarium of Duke-NUS medical school, Singapore. Male littermate mice (8-12 weeks) were assigned to the WT and Mut/Mut groups according to the results of genotyping.
The WWP2flox/flox mice were generated based on C57BL/6J in Shanghai Model Organisms and B6.129P2-Lyz2tm1(cre)Ifo/J (#004781) from the Jackson Laboratory. The WWP2Mac (WWP2flox/floxLyzcre) mice were crossbreed with WWP2flox/flox and B6.129P2-Lyz2tm1(cre)Ifo/J in the vivarium of Duke-NUS medical school, Singapore. Male littermate mice (8-12 weeks) were assigned to the WT and WWP2Mac groups according to the results of genotyping.
Mice were bred and maintained in animal facility at Duke-NUS medical school prior to use. All mice were housed in a specific pathogen–free (SPF) environment and complied with all relevant ethical regulations according to guidelines issued by the National

	Advisory Committee on Laboratory Animal Research. The housing room was set to a 12 h light/dark cycle with lights off at 8 a.m., a temperature of about 22°C and a relative air humidity of about 50%. Protocol with IACUC number 2016/SHS/1170 was approved by Institutional Animal Care and Use Committee of National University of Singapore, Duke-NUS Medical School. Steps were performed to minimize animal suffering according to guidelines of the Singhealth Council on Animal Care.
Wild animals	The study did not involve wild animals.
Field-collected samples	The study did not involve field-collected samples.
Ethics oversight	All relevant ethical regulations according to guidelines was issued by the National Advisory Committee on Laboratory Animal Research. Protocol with IACUC number 2016/SHS/1170 was approved by Institutional Animal Care and Use Committee of National University of Singapore, Duke-NUS Medical School.

Note that full information on the approval of the study protocol must also be provided in the manuscript.

Flow Cytometry

Plots

Confirm that:

☒ The axis labels state the marker and fluorochrome used (e.g. CD4-FITC).

☒ The axis scales are clearly visible. Include numbers along axes only for bottom left plot of group (a 'group' is an analysis of identical markers).

☒ All plots are contour plots with outliers or pseudocolor plots.

☒ A numerical value for number of cells or percentage (with statistics) is provided.

Methodology

Sample preparation	Mouse heart was perfused, excised, minced, digested with Collagenase II (#LS004174, Worthington Biochemical Corporation) and Dispase II (#494207800, Roche). Tissue mixture was mechanically disrupted and filtered through 70 µm cell strainer to get single cell suspension. After blocking with CD16/32 (#14-0161-85,Thermo fisher, 1:100) at RT for 10 min.
Instrument	cells were collected by centrifugation, subjected to live-death dye and surface antibody staining, and analysed or sorted by flow cytometry using BD FACS ARIA system.
Software	BD FACS ARIA system and FlowJo 10 were used for FACS data analysis
Cell population abundance	For each sample, a total of 500,000 cells were captured. After filtration of cells out of bounds with adjusted voltage, the relevant population for within post-sort fractions were around 1,000, which is abound for further analysis.
Gating strategy	Cardiac macrophages, mocncytes or subpopulation were gated from whole heart cells using surface antibodies using CD45 +CD64+CD11b+F4/80+Ly6G- or CD45+CD64+CD11b+/Ly6Chigh, respectively. The detail antibodies used as following: Anti-Ly-6C Monoclonal Antibody PerCP-Cyanine5.5 (#45-5932-82, Thermo fisher, 1:25), APC Rat Anti-CD11b (#561690,1:100, BD Biosciences), FITC Mouse Anti-Mouse CD45 (#561874, 1:100, BD Biosciences), APC-H7 Rat Anti-Mouse Ly-6G (#565369, 1:200, BD Biosciences), PE Mouse anti-Mouse CD64 (#558455, 1:100, BD Biosciences), BV421 Rat Anti-Mouse F4/80 (#565411, 1:100, BD Biosciences). The detail gate strategy is provide in Supplementary Figure 1 and 4.

☒ Tick this box to confirm that a figure exemplifying the gating strategy is provided in the Supplementary Information.
