# Supplementary material for: The E3 ubiquitin ligase WWP2 regulates pro-fibrogenic monocyte infiltration and activity in heart fibrosis
Source: Nat Commun. 2022 Nov 30;13:7375. doi: 10.1038/s41467-022-34971-6 (PMC9712659; doi:10.1038/s41467-022-34971-6)

Figure 2i

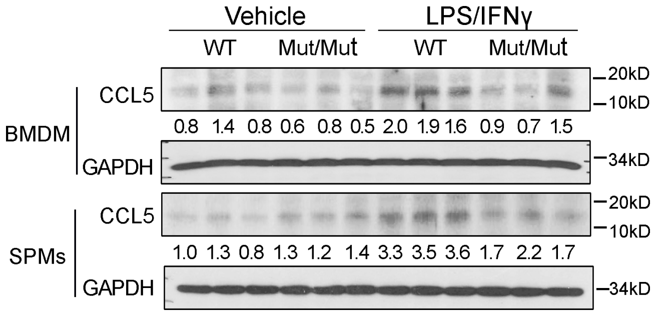

Original blots

Ccl5 Blot In BMDM

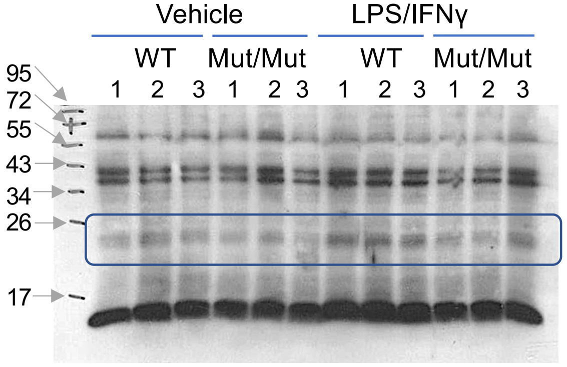

GAPDH Blot (Ccl5)

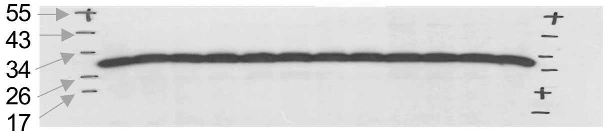

Ccl5 Blot In Spleen derived macrophages

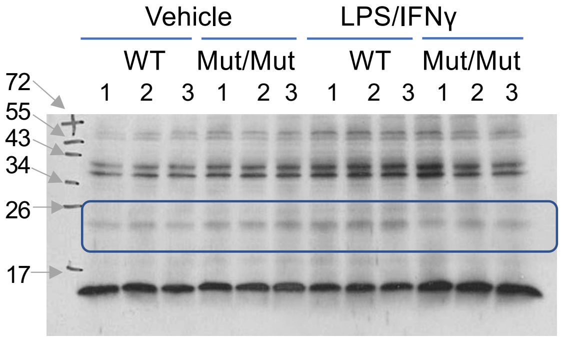

GAPDH Blot (Ccl5)

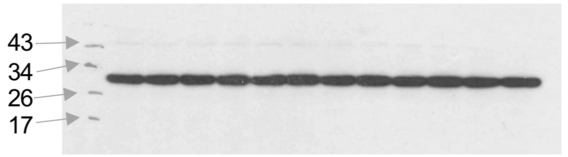

Figure 3i

Original blots

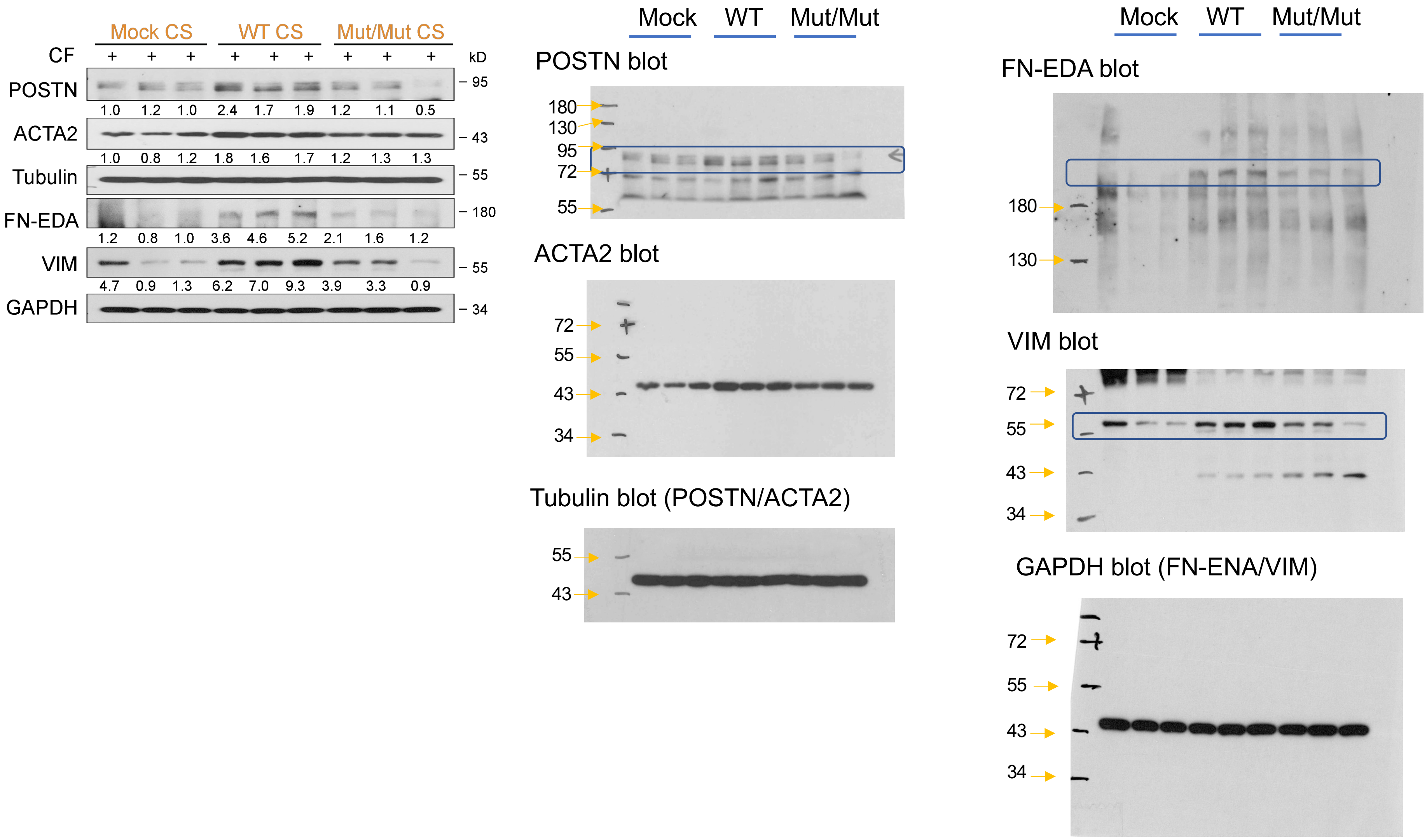

Figure 4e

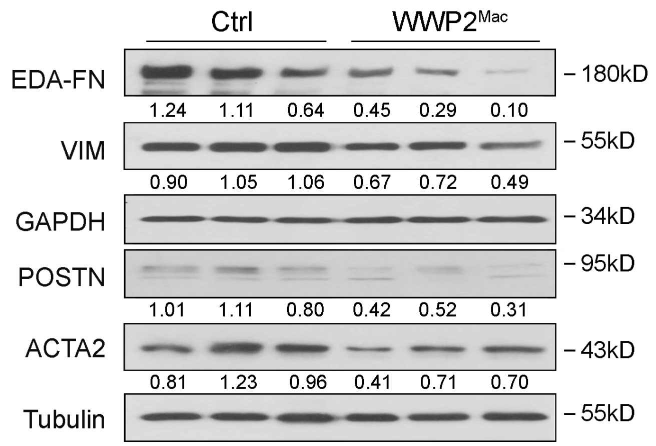

Original blots

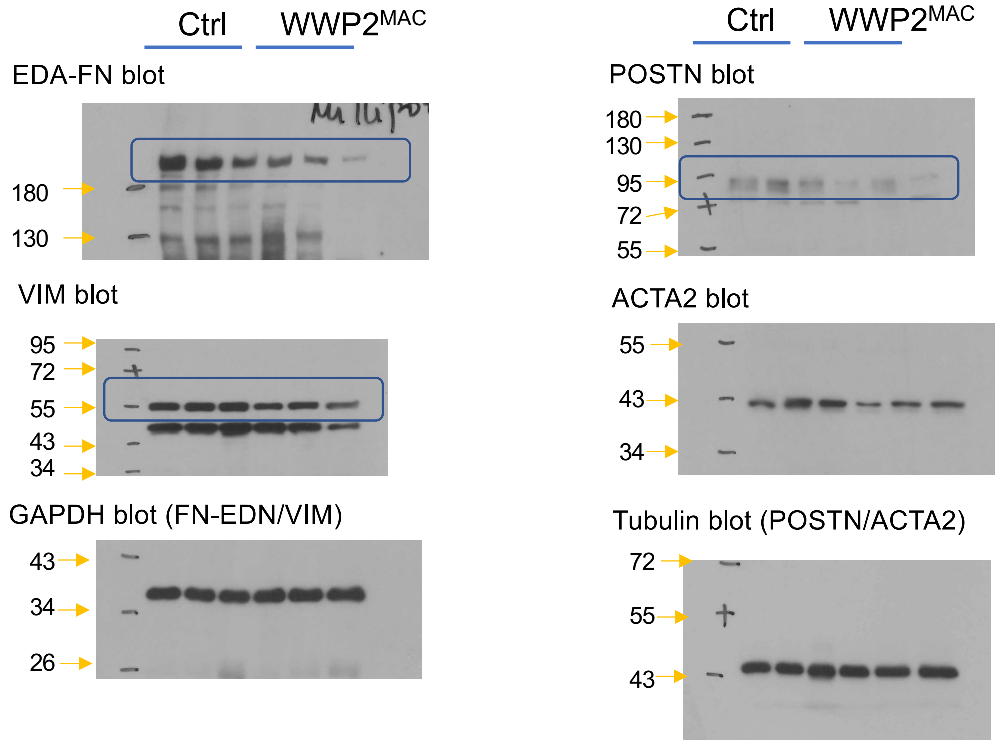

Figure 4j down

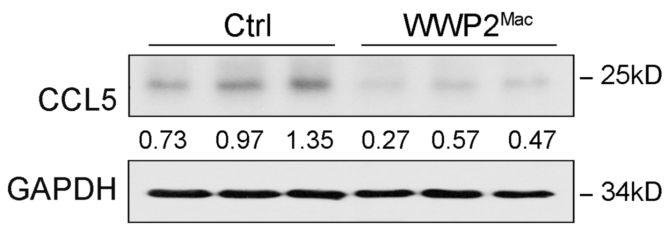

Original blots

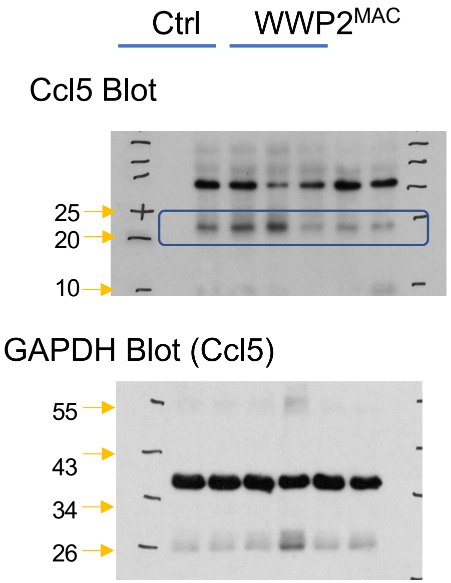

Figure 4m

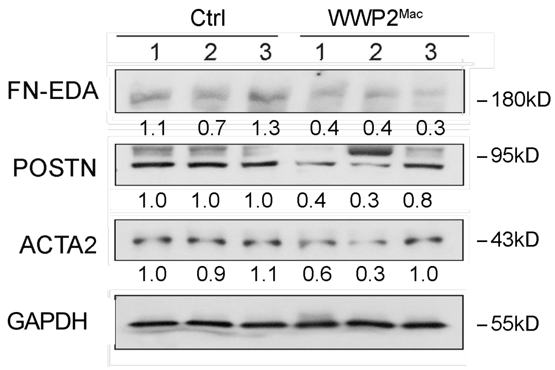

Original blots

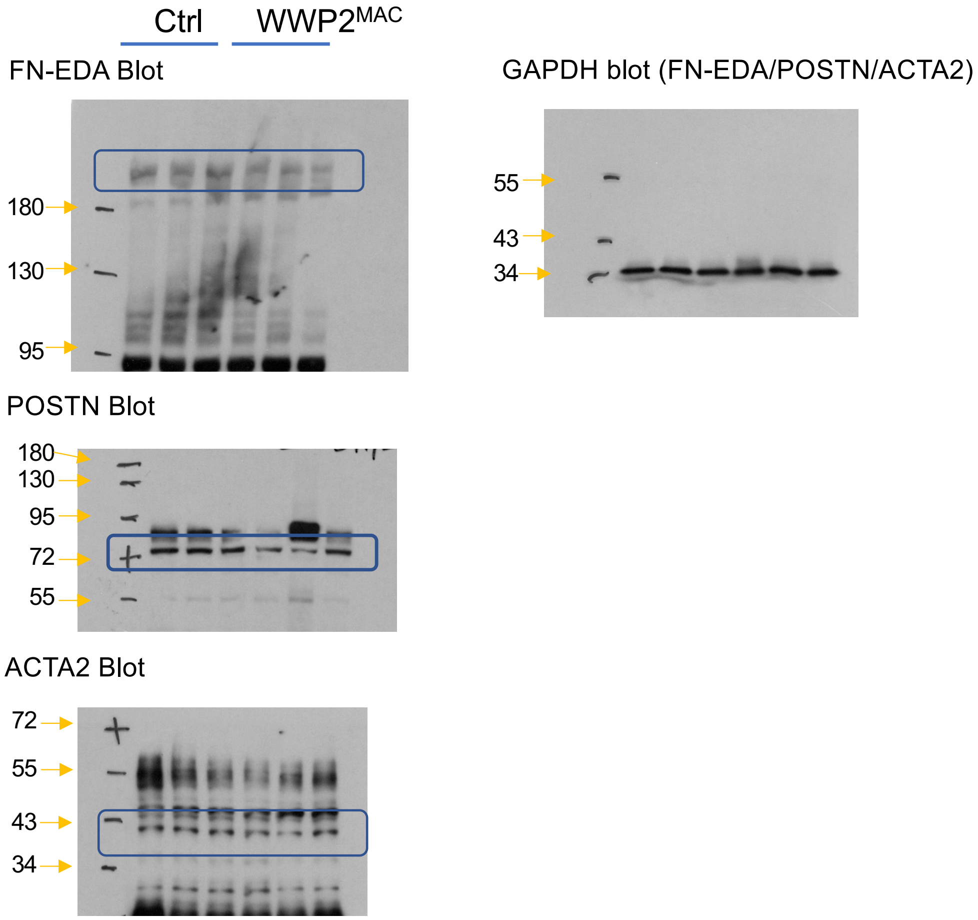

Figure 5e

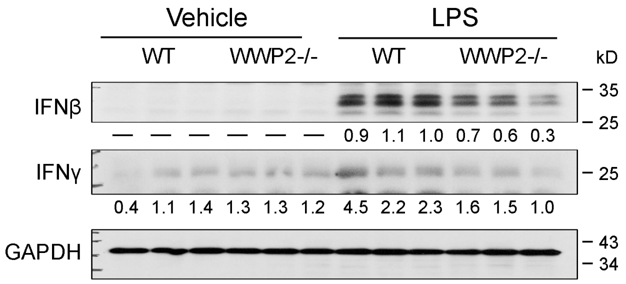

Original blots

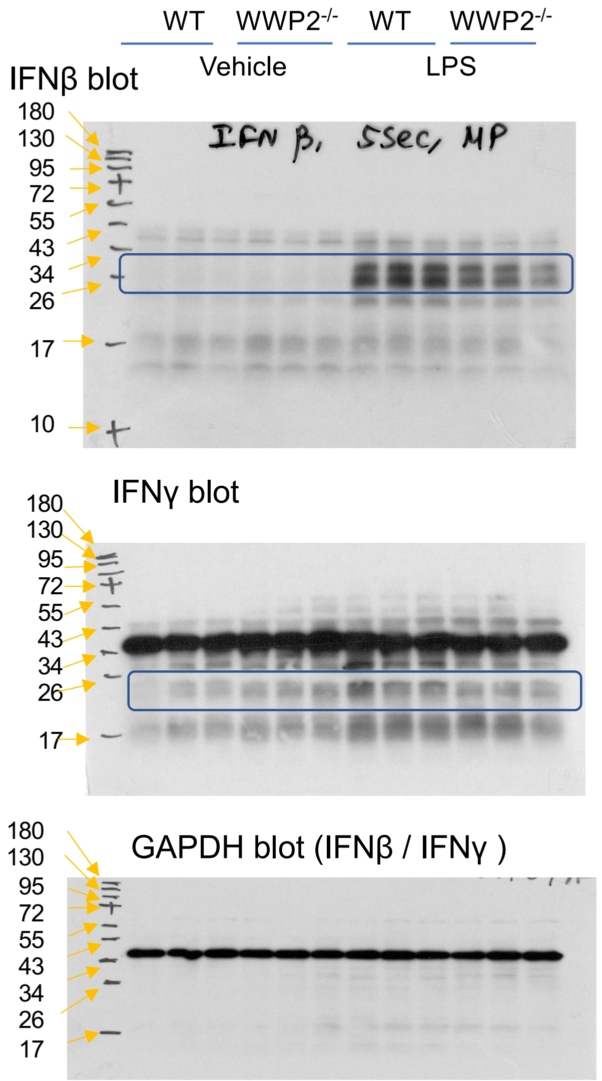

Figure 5i

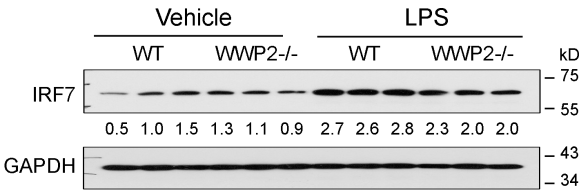

Original blots

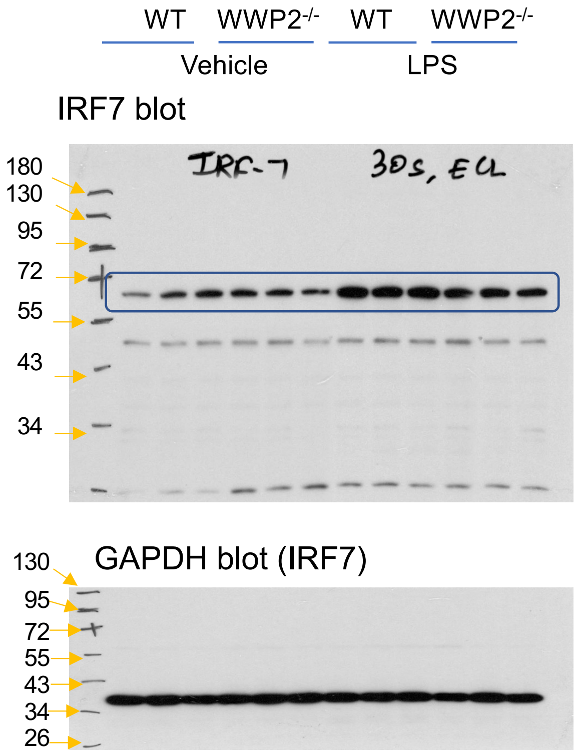

Figure 6a upper

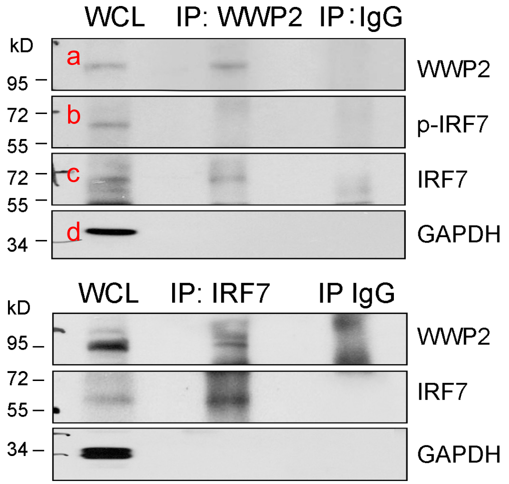

Original blots

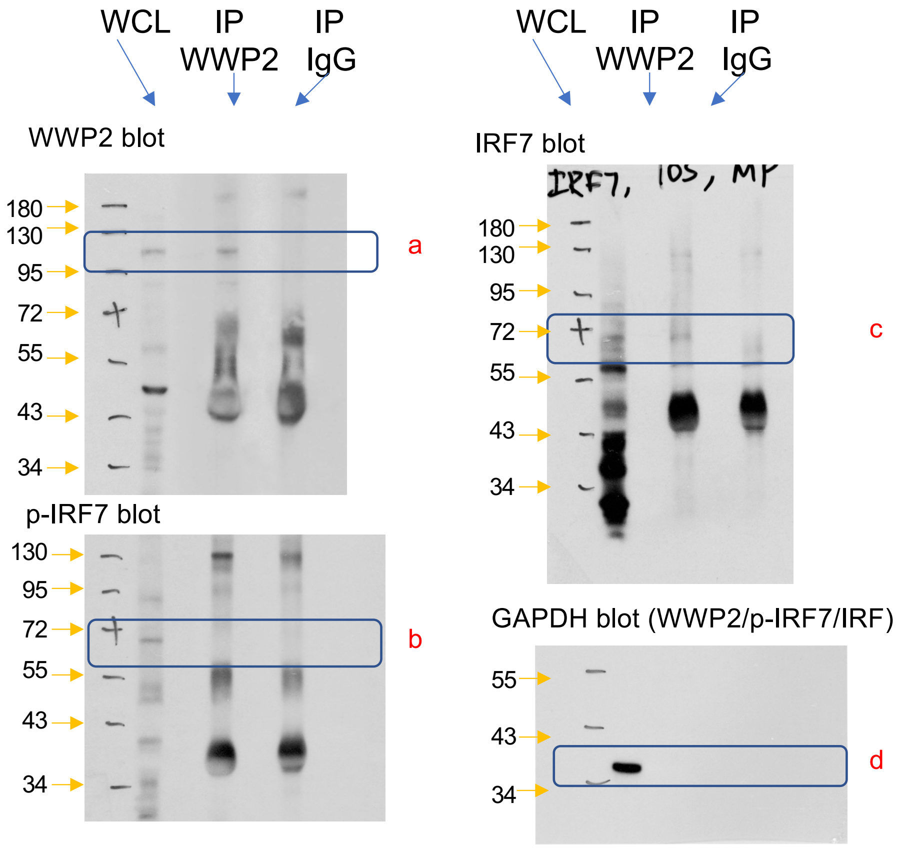

Figure 6a down

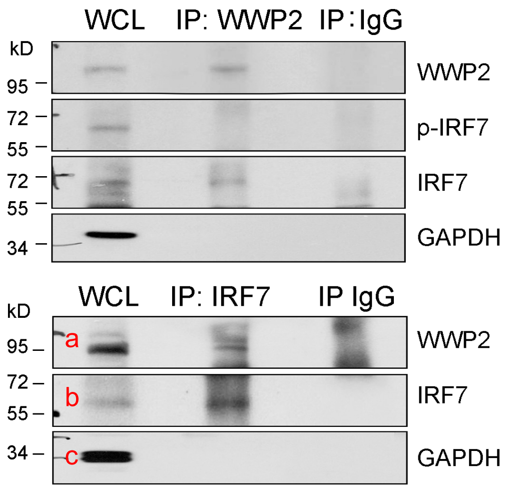

Original blots

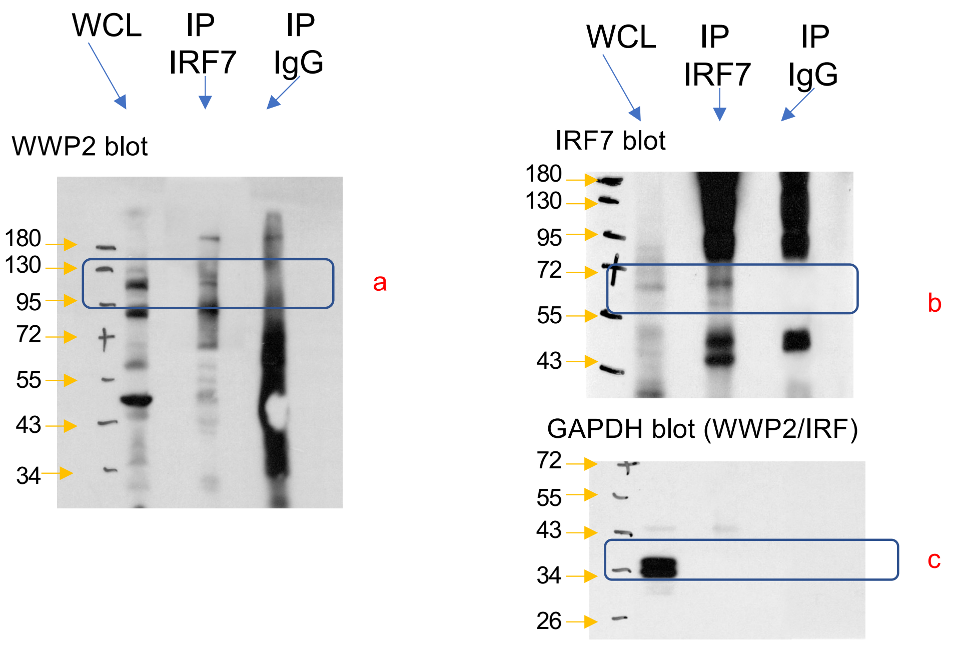

Figure 6c

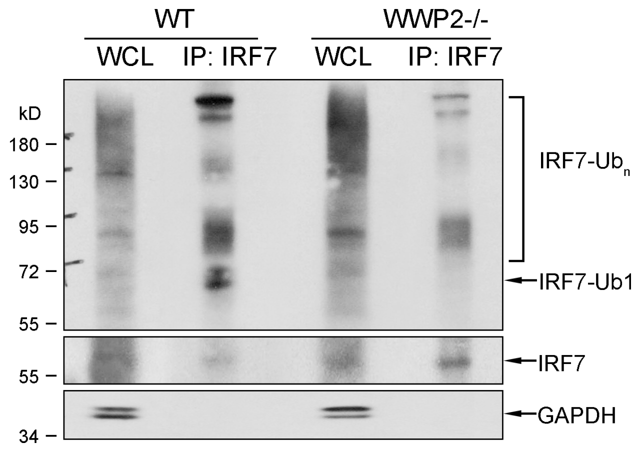

Original blots

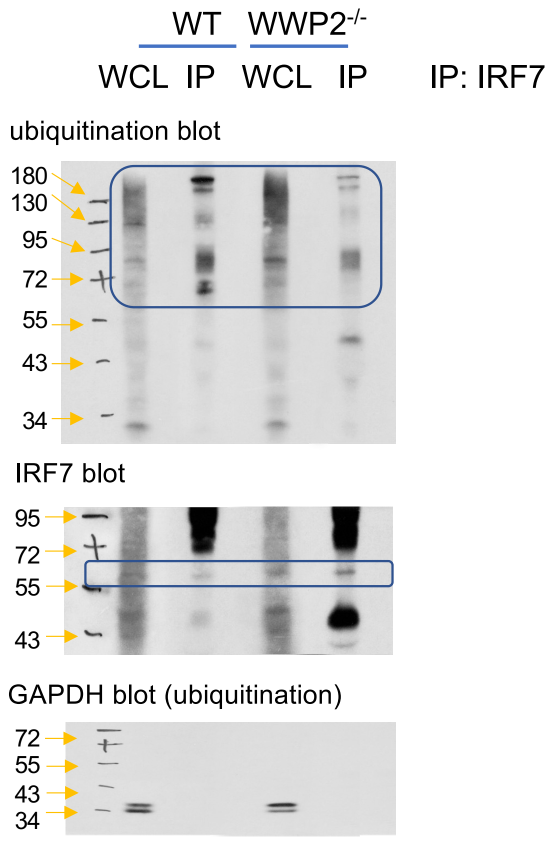

Figure 6e

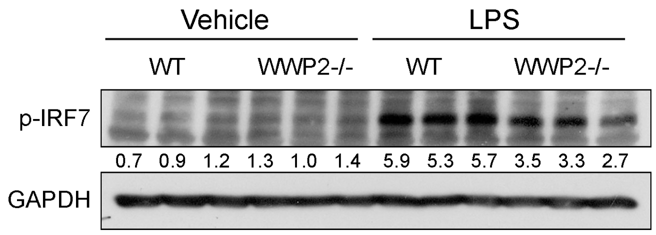

Original blots

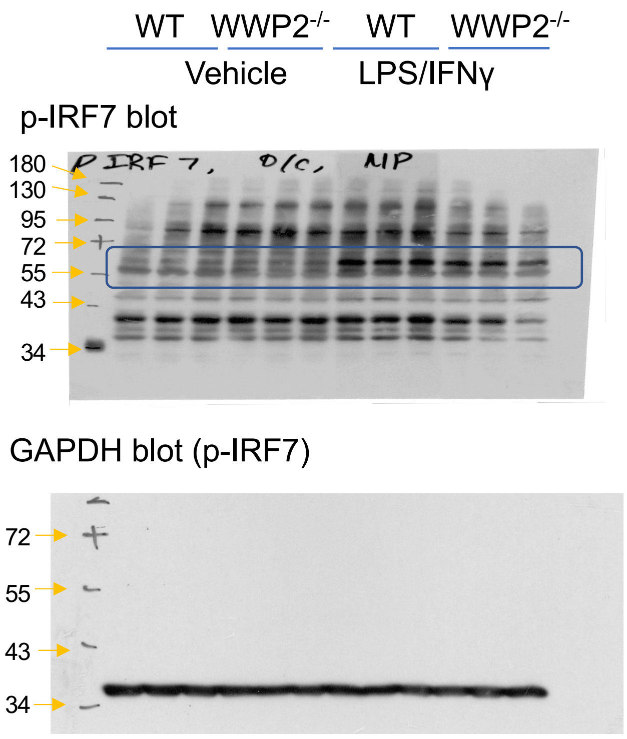

Figure 6f

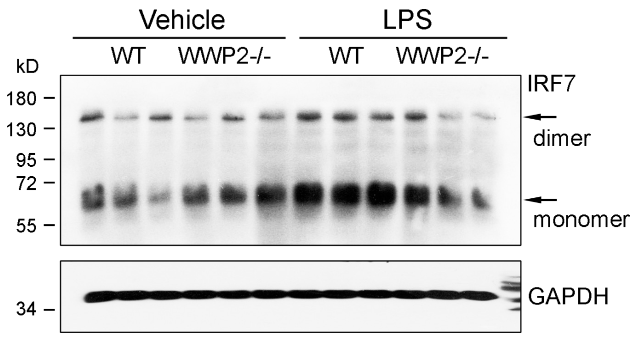

Original blots

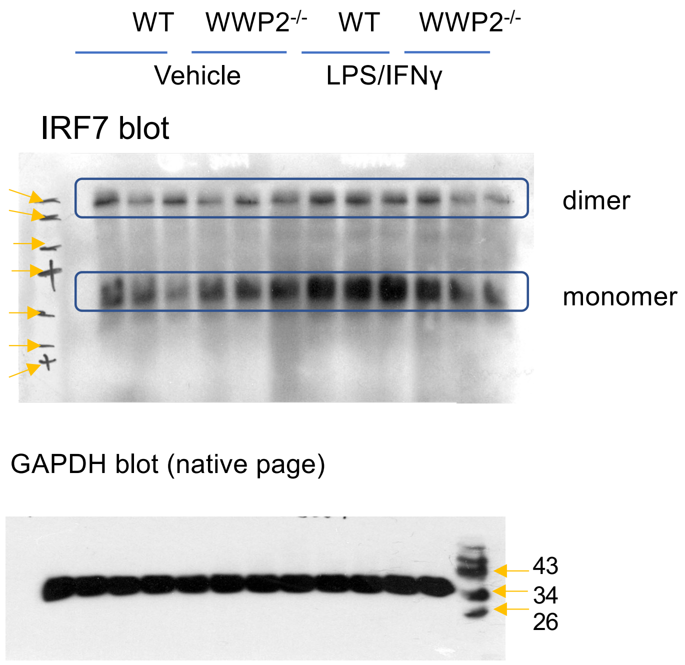

Figure 6j

Nuclear Fraction

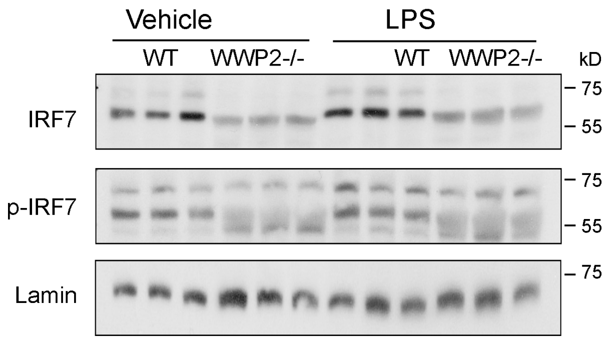

Original blots nuclear extraction

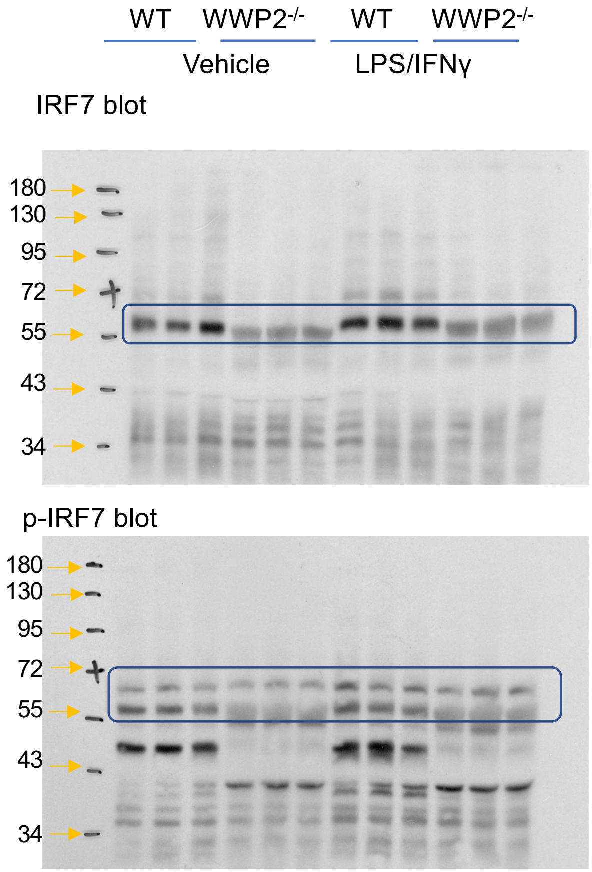

Lamin blot (IRF7/p-IRF7)

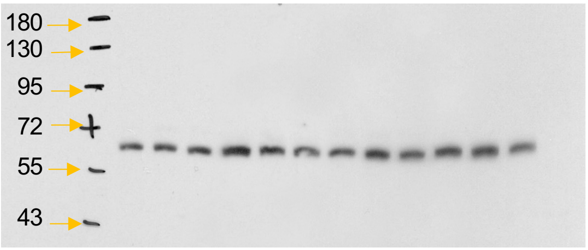

Supplementary Figure 11b

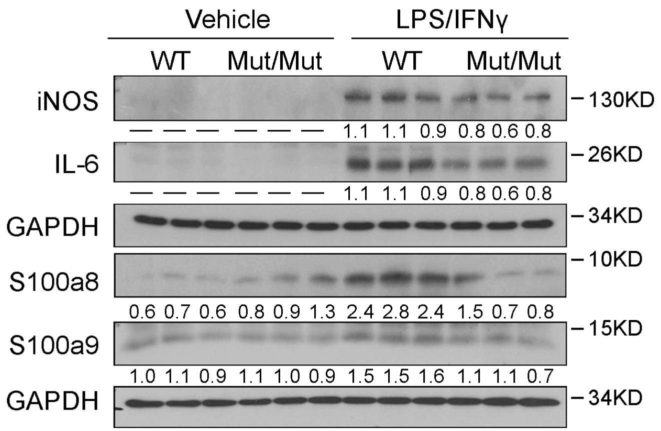

Original blots

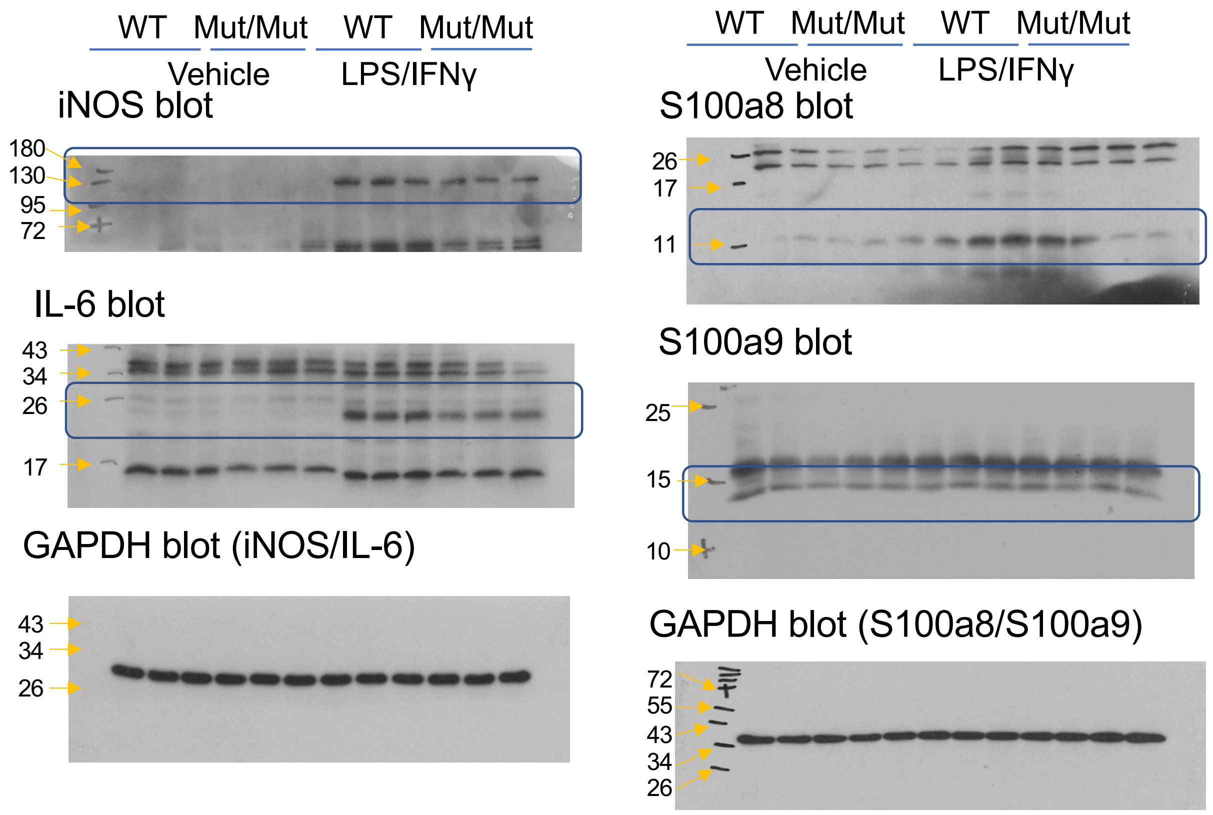

Supplementary Figure 12a

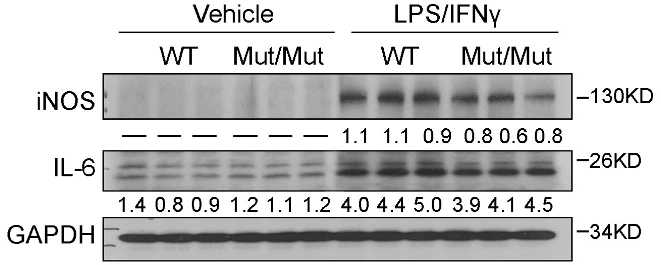

Original blots

WT Mut/Mut WT Mut/Mut
Vehicle LPS/IFNγ

iNOS blot

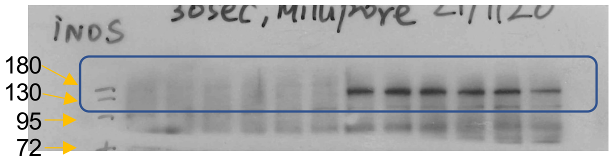

IL-6 blot

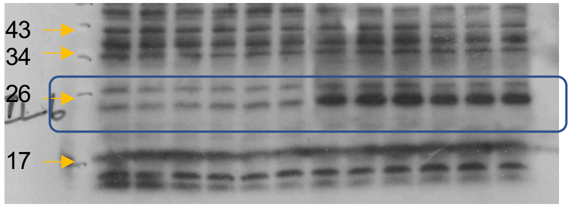

GAPDH blot (iNOS/IL-6)

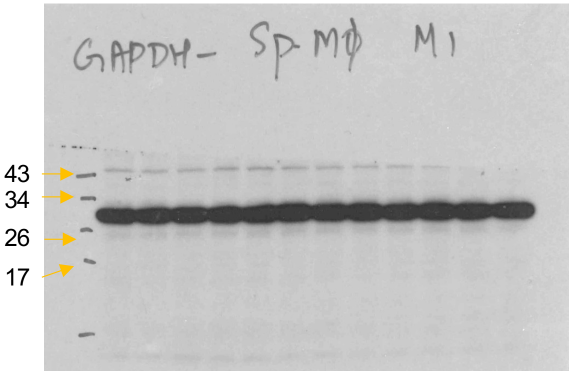

Supplementary Figure 15b

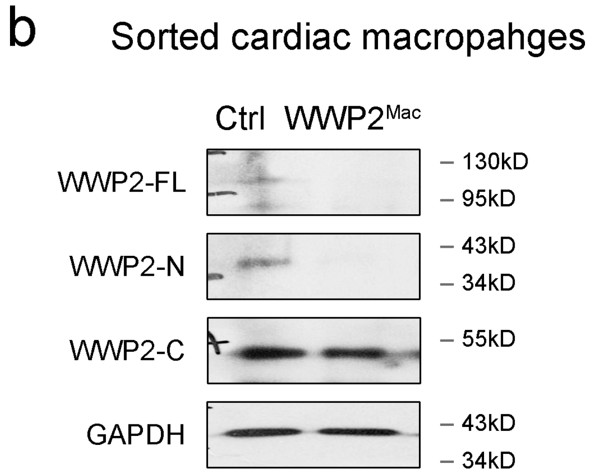

Original blots

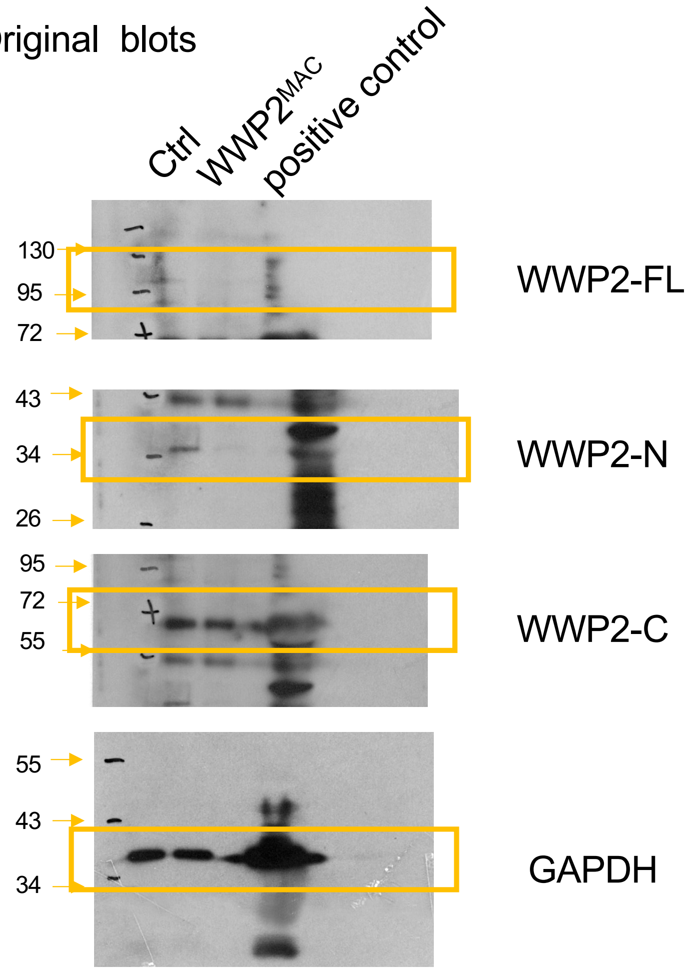

Supplementary Figure 15c

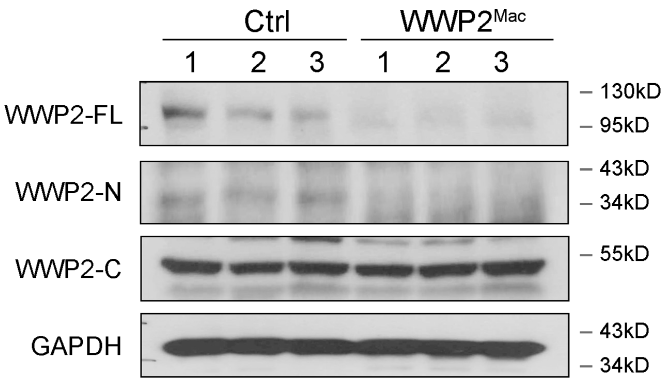

Ctrl: WWP2$^{f/f}$LysM$^{-/-}$

WWP2^{Mac}: WWP2$^{f/f}$LysM$^{Cre/-}$

Original blots

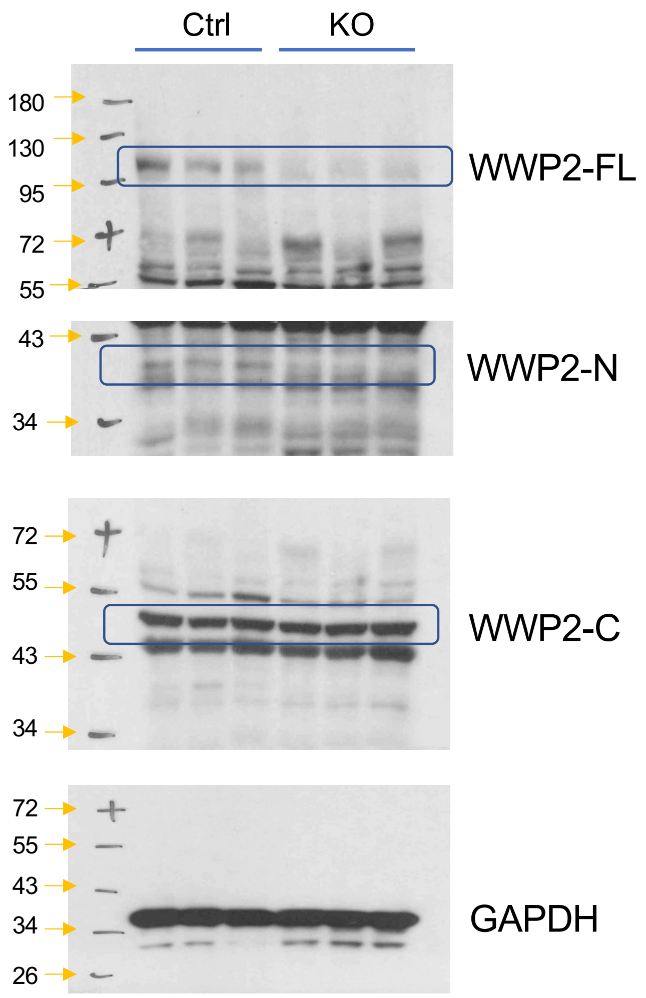

Supplementary Figure 15d

Original blots

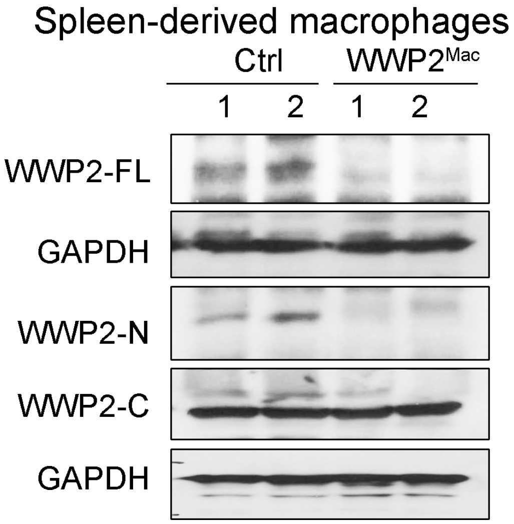

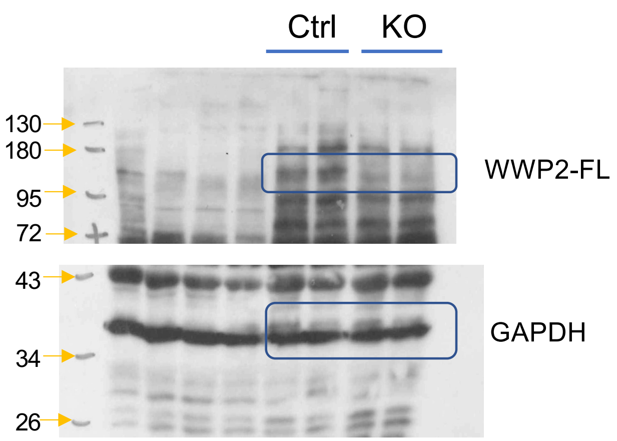

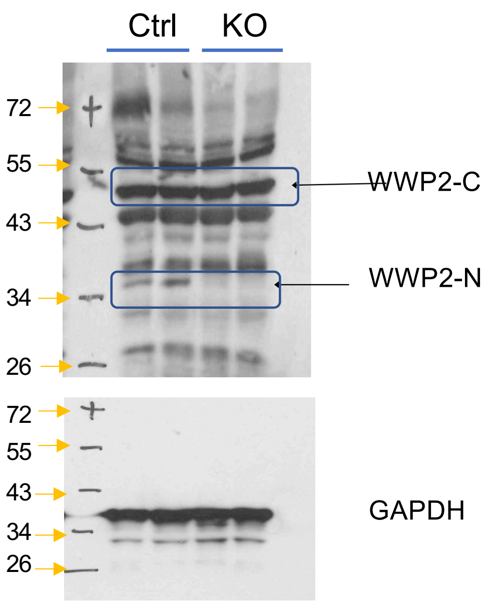

Supplementary Figure 15e

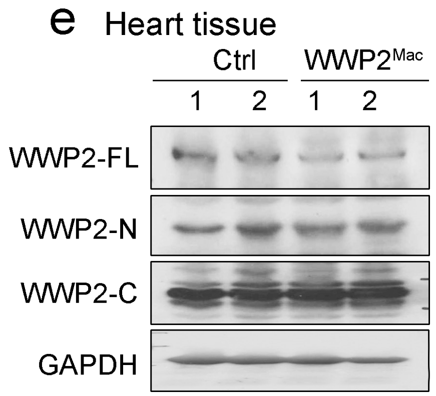

Original blots

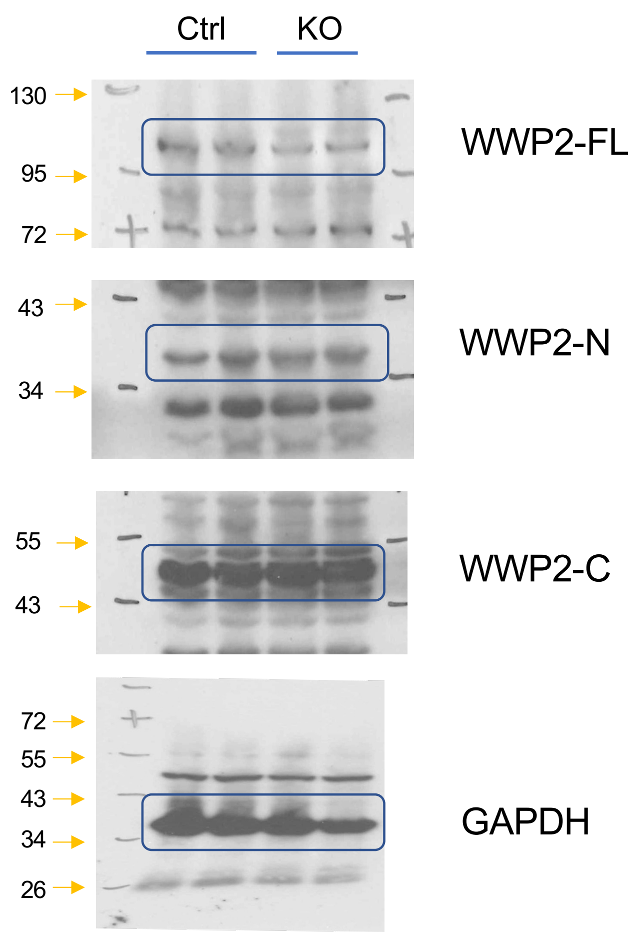

Supplementary Figure 15f

Cardiac fibroblasts

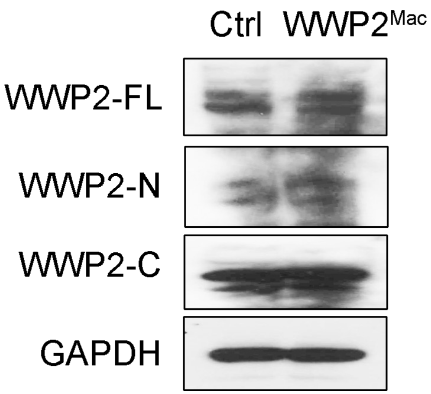

Original blots

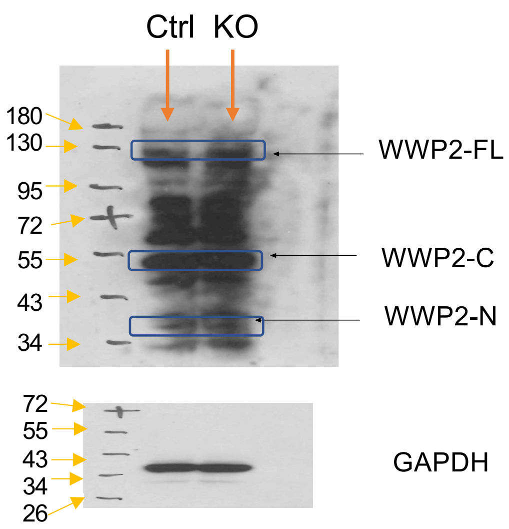

Supplementary Figure 18a

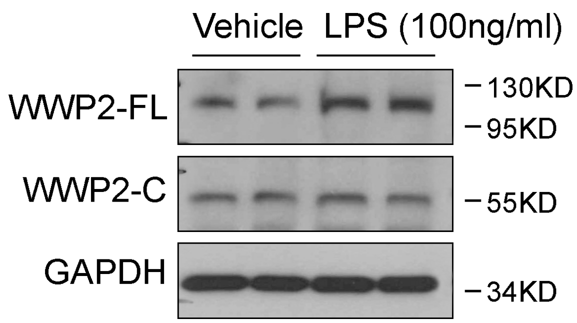

Original blots

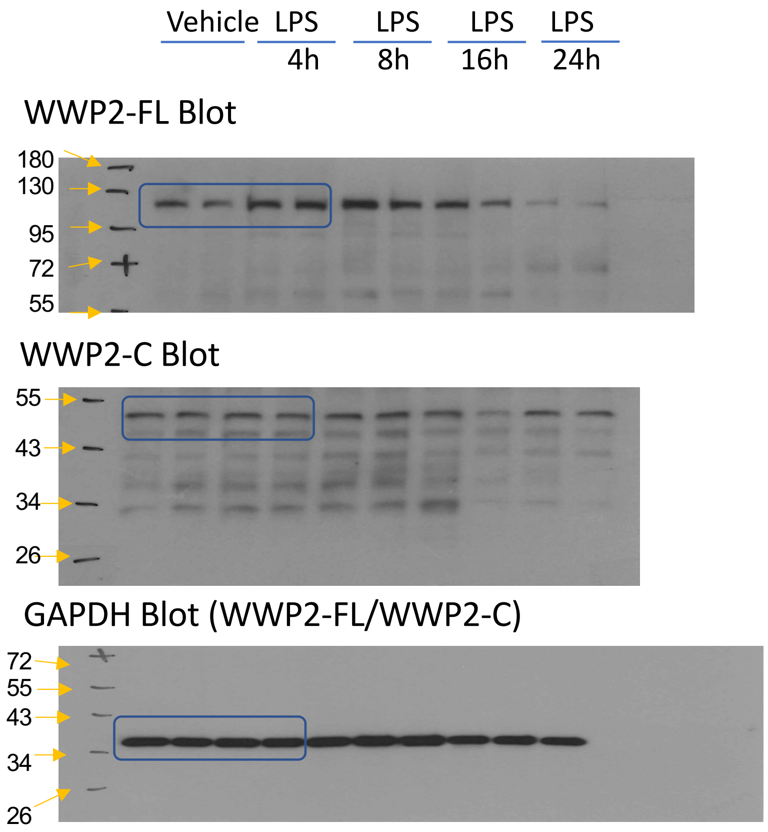

Supplementary Figure 18e

c

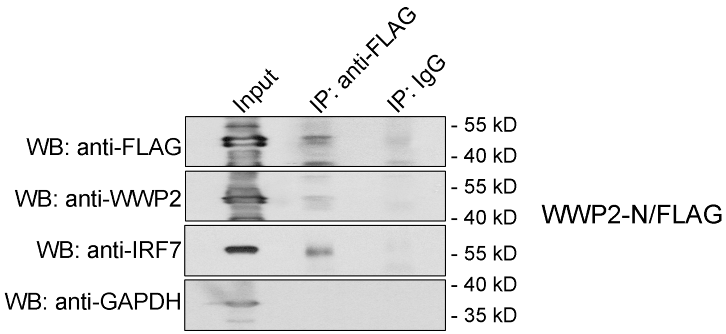

Original blots

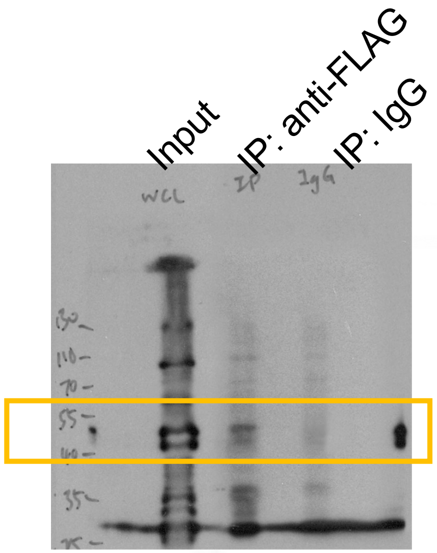

FLAG blot

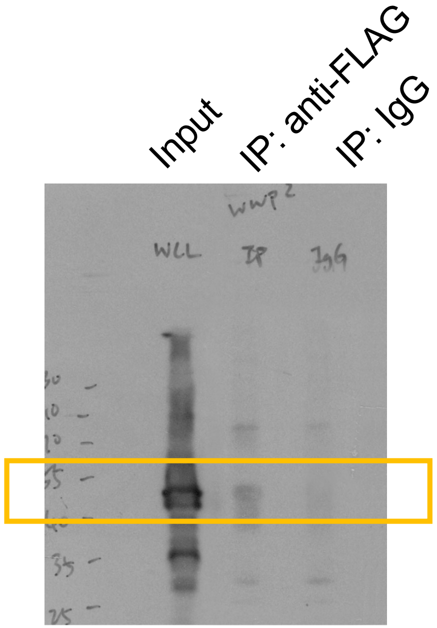

WWP2 blot

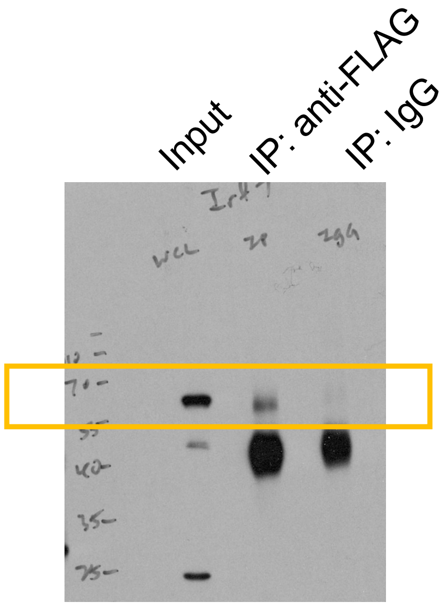

IRF7 blot

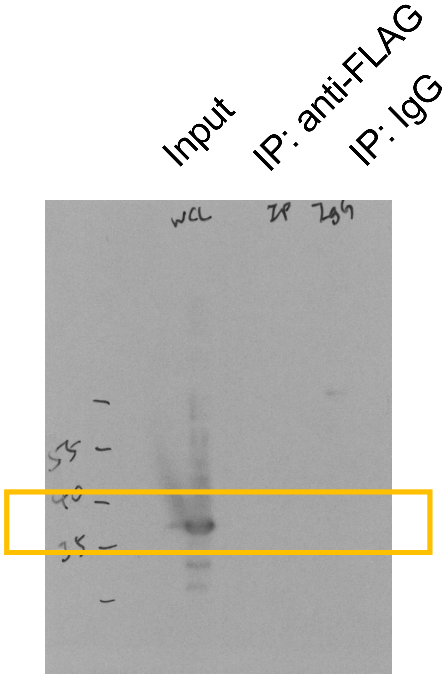

GAPDH blot

Supplementary Figure 18e

c

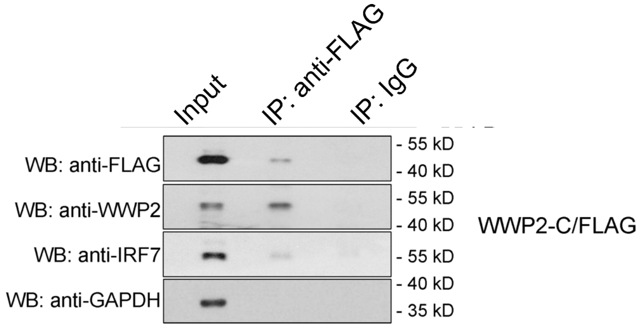

Original blots

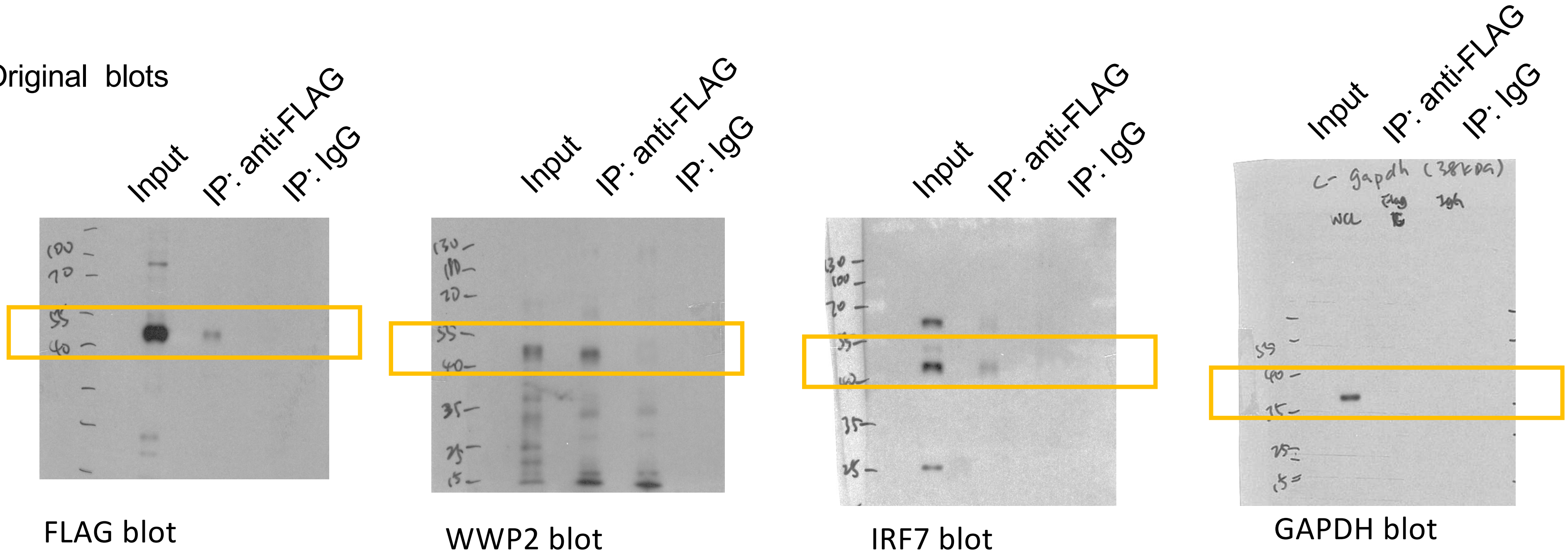

Supplementary Figure 18e

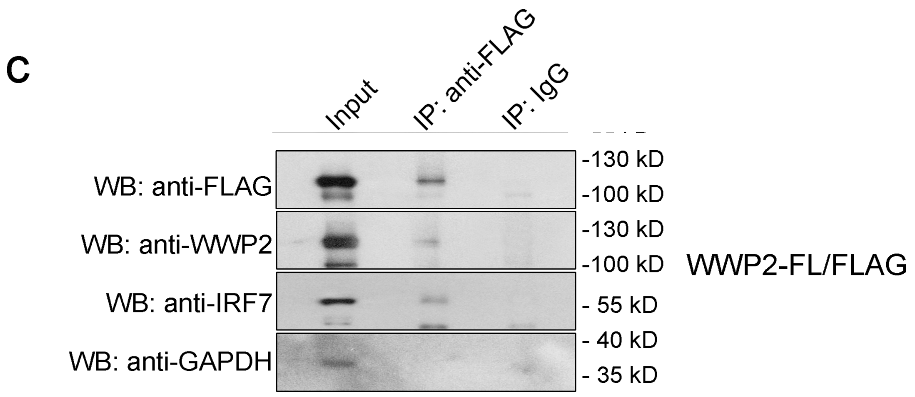

Original blots

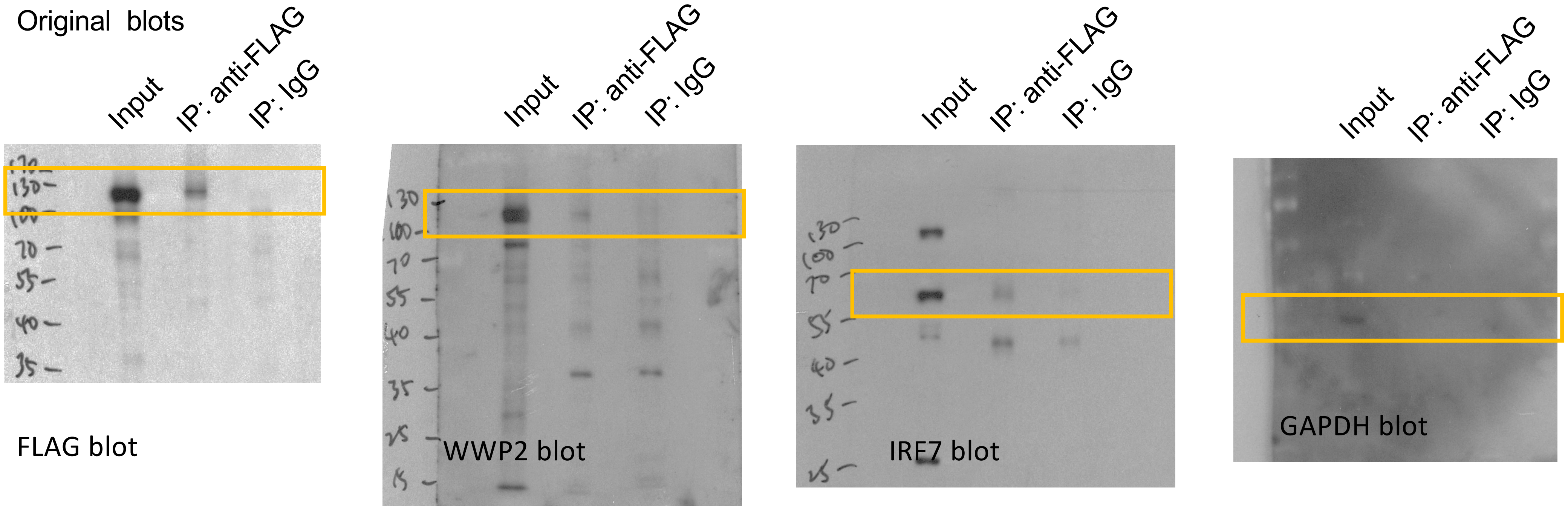

Supplementary Figure 19a

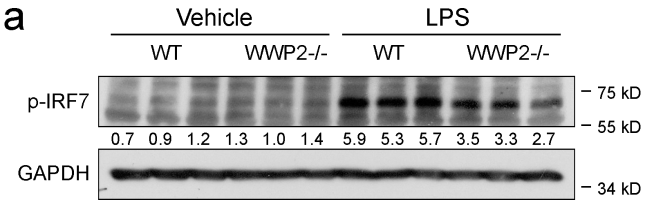

Original blots

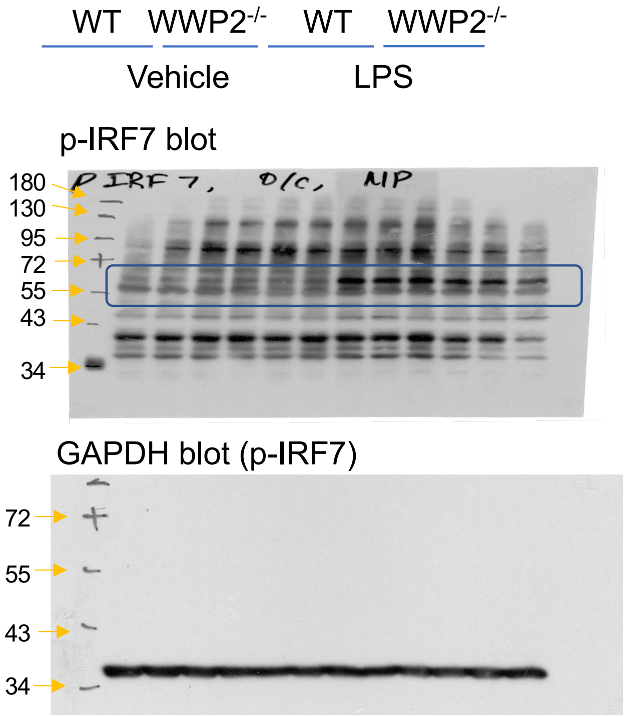

Supplement: Supplementary file 4 — Source Data [file 41467_2022_34971_MOESM4_ESM.zip › 359229_2_data_set_7039930_rkm3v3.pdf]
